# Deregulation of mTORC1-TFEB axis in human iPSC model of *GBA1*-associated Parkinson’s disease

**DOI:** 10.3389/fnins.2023.1152503

**Published:** 2023-06-02

**Authors:** Fahad Mubariz, Afsoon Saadin, Nicholas Lingenfelter, Chinmoy Sarkar, Aditi Banerjee, Marta M. Lipinski, Ola Awad

**Affiliations:** ^1^Department of Microbiology and Immunology, University of Maryland School of Medicine, Baltimore, MD, United States; ^2^Department of Anesthesiology, University of Maryland School of Medicine, Baltimore, MD, United States; ^3^Department of Pediatrics, University of Maryland School of Medicine, Baltimore, MD, United States; ^4^Department of Anatomy and Neurobiology, University of Maryland School of Medicine, Baltimore, MD, United States

**Keywords:** *GBA1* mutations, Parkinson’s disease, transcription factor EB, induced-pluripotent stem cells, autophagy-lysosomal pathway, mammalian target of rapamycin complex1

## Abstract

Mutations in the *GBA1* gene are the single most frequent genetic risk factor for Parkinson’s disease (PD). Neurodegenerative changes in *GBA1*-associated PD have been linked to the defective lysosomal clearance of autophagic substrates and aggregate-prone proteins. To elucidate novel mechanisms contributing to proteinopathy in PD, we investigated the effect of *GBA1* mutations on the transcription factor EB (TFEB), the master regulator of the autophagy-lysosomal pathway (ALP). Using PD patients’ induced-pluripotent stem cells (iPSCs), we examined TFEB activity and regulation of the ALP in dopaminergic neuronal cultures generated from iPSC lines harboring heterozygous *GBA1* mutations and the CRISPR/Cas9-corrected isogenic controls. Our data showed a significant decrease in TFEB transcriptional activity and attenuated expression of many genes in the CLEAR network in *GBA1* mutant neurons, but not in the isogenic gene-corrected cells. In PD neurons, we also detected increased activity of the mammalian target of rapamycin complex1 (mTORC1), the main upstream negative regulator of TFEB. Increased mTORC1 activity resulted in excess TFEB phosphorylation and decreased nuclear translocation. Pharmacological mTOR inhibition restored TFEB activity, decreased ER stress and reduced α-synuclein accumulation, indicating improvement of neuronal protiostasis. Moreover, treatment with the lipid substrate reducing compound Genz-123346, decreased mTORC1 activity and increased TFEB expression in the mutant neurons, suggesting that mTORC1-TFEB alterations are linked to the lipid substrate accumulation. Our study unveils a new mechanism contributing to PD susceptibility by *GBA1* mutations in which deregulation of the mTORC1-TFEB axis mediates ALP dysfunction and subsequent proteinopathy. It also indicates that pharmacological restoration of TFEB activity could be a promising therapeutic approach in *GBA1*-associated neurodegeneration.

## Introduction

Mutations in the *GBA1* gene, which encodes the lysosomal enzyme Glucocerebrosidase (GCase) are the most frequent genetic risk factor for both familiar and sporadic Parkinson’s disease (PD) (Siebert et al., 2014; Schapira, 2015). GCase is a lysosomal hydrolase responsible for the breakdown of the cellular sphingolipids; glucosylceramide (GlcCer) and glucosylsphingosine (GlcSph) (Boer et al., 2020). Reduced GCase enzyme activity results in pathogenic accumulation of its substrates and subsequent disruption of cellular lipid homeostasis (Xu et al., 2010; Navarro-Romero et al., 2022). Biallelic *GBA1* mutations cause Gaucher’s Disease (GD), the most common lysosomal storage disorder (Mignot et al., 2013). GD is characterized by a wide spectrum of neurological manifestations and progressive neurodegeneration in its severe forms (Vitner and Futerman, 2013; Roshan Lal and Sidransky, 2017). The risk of developing PD among GD patients is more than twenty times that of the general population (Swan and Saunders-Pullman, 2013). *GBA1* mutations are associated with not only an increased risk of PD but also early disease onset and severe course (Sidransky, 2012; Do et al., 2019). Reduced GCase activity has been also reported in the substantia nigra of PD patients without *GBA1* mutations, indicating the relevance of GCase functions to PD in general (Rocha et al., 2015). Accumulating evidence from experimental models of PD and patient samples indicated a correlation between decreased GCase enzyme activity and accumulation of α-synuclein, the major constituent of Lewy bodies and the hallmark of PD (Behl et al., 2021; Gegg et al., 2022). Reduced GCase activity and subsequent accumulation of its lipid substrates are shown to affect the trafficking, processing, and clearance of α-synuclein thus favoring its toxic accumulation in the midbrains of affected subjects (Bae et al., 2015; Mazzulli et al., 2016; Navarro-Romero et al., 2022).

In *GBA1*-associated PD, lysosomal dysfunction and perturbation of lipid homeostasis appear to play a central role in driving the disease process (Do et al., 2019; Glajch et al., 2021). The autophagy-lysosomal pathway (ALP) is responsible for the clearance of defective cellular organelles and aggregate-prone proteins, which is critical for neuronal survival (Harris and Rubinsztein, 2011; Menzies et al., 2017). *GBA1* mutations are known to cause lysosomal alterations and autophagic perturbations in various animal and cellular models (Sun et al., 2010; Sun and Grabowski, 2010; Sanchez-Martinez et al., 2016). Studies using human induced-pluripotent stem cells (iPSCs) harboring *GBA1* mutations reported ALP dysfunction in dopaminergic (DA) neurons from PD patients and further highlighted its role in PD-associated proteinopathy (Schondorf et al., 2014; Fernandes et al., 2016; Mazzulli et al., 2016). Inhibition of macroautophagy (refers to as autophagy afterward), decreased autophagosome-lysosome fusion, and lower Cathepsin D levels and activity, were all detected in PD patients’ DA neurons and were shown to favor α-synuclein accumulation (Lehtonen et al., 2019; Navarro-Romero et al., 2020; Yang et al., 2020).

We previously demonstrated that ALP alterations in neuronopathic GD neurons harboring biallelic *GBA1* mutations are linked to the deregulation of the transcription factor EB (TFEB), the master regulator of the lysosomal and autophagy genes (Awad et al., 2015; Brown et al., 2019). TFEB controls the ALP gene expression through binding to the Coordinated Lysosomal Expression and Regulation (CLEAR) motif in their promoters (Settembre et al., 2011). TFEB is phosphorylated by a set of cellular kinases, which modulate its nuclear translocation and transcriptional activity in response to cellular energy demands (Settembre et al., 2012; Puertollano et al., 2018). Activation of TFEB induces the expression of genes required for lysosomal biogenesis, autophagosome formation and fusion, and substrate degradation thus enhancing the ALP functions (Rai et al., 2021). It has been shown that TFEB is widely expressed in the CNS and exerts a neuroprotective role mainly by preventing protein aggregation (Decressac et al., 2013; Gu et al., 2022). Impaired TFEB localization and activity have been detected in many neurodegenerative diseases including PD, and were linked to the defective neuronal clearance of autophagic substrates and aggregate-prone proteins (Decressac et al., 2013; Cortes and La Spada, 2019). Our previous work uncovered that both TFEB levels and activity are reduced in neuronopathic GD neurons, which resulted in lysosomal depletion and autophagy block (Awad et al., 2015). We further showed that ALP alterations in those mutant neurons are mediated by hyperactivity of the mammalian target of rapamycin complex1 (mTORC1), the major cellular metabolic sensor and the key negative regulator of TFEB activity (Napolitano et al., 2018; Brown et al., 2019). These results led us to hypothesize that *GBA1*-mediated deregulation of TFEB activity might be involved in promoting proteinopathy in PD.

Previous studies reported altered TFEB protein expression in post-mortem PD patients’ brains and showed a significant reduction in its level in the nuclear compartment of the nigral DA neurons (Decressac and Bjorklund, 2013). TFEB is also shown to interact with α-synuclein, which sequesters it in the cytosol, thus preventing its nuclear translocation and activity (Decressac et al., 2013). Similarly, a high lipid load is shown to retain TFEB in the cytosol, suggesting that pathogenic accumulation of cellular lipids can diminish TFEB activity (Settembre et al., 2013). *GBA1* mutations are shown to cause lipid substrate accumulation in PD neurons (Schondorf et al., 2014; Huebecker et al., 2019), which may cause subsequent alterations in TFEB regulation and activity. In support of this idea, it was found that expression of wild-type *GBA1* successfully reduced lipid-rich α-synuclein aggregates and increased nuclear TFEB translocation in a PD mouse model (Glajch et al., 2021). Another potential mechanism contributing to TFEB dysfunction in neurodegenerative disorders is the disruption of its upstream regulatory signals by major cellular kinases such as mTORC1 and Glycogen synthase kinase-3β (GSK3β) (Parr et al., 2012; Cortes and La Spada, 2019). Increased levels of mTOR, have been previously reported in postmortem brains of PD patients and patients displaying α-synuclein accumulation (Wills et al., 2012). Furthermore, pharmacological or genetic inhibition of mTOR activity is shown to improve autophagic clearance, reverse DA neuronal damage and alleviate PD symptoms (Jahrling and Laberge, 2015; Martini-Stoica et al., 2016). Hence, accumulating evidence points to the potential involvement of the deregulation in the mTOR-TFEB axis in PD development and progression.

Enhancement of TFEB activity is shown to promote protein clearance and neuroprotection in PD animal models, which makes it a promising therapeutic target (Decressac and Bjorklund, 2013; Sardiello, 2016). However, the effect of PD-associated mutations on endogenous TFEB regulation and activity remains unclear. In this study, we investigated the effect of *GBA1* mutations, the most common genetic risk factor for PD on TFEB transcriptional activity and regulation of the ALP in dopaminergic neuronal cultures (DNCs) generated from PD patients’ iPSC lines. Our data showed a significant decrease in TFEB activity and attenuated expression of many genes in the CLEAR network in PD DNCs harboring *GBA1* mutations. We also found increased TFEB phosphorylation and decreased nuclear translocation in the mutant neurons. Importantly, TFEB alterations were not present in PD neurons generated from the isogeneic gene-corrected iPSC lines, indicating that these changes are mediated by *GBA1* mutations. Consistent with increased TFEB phosphorylation, we found a significant increase in mTORC1 kinase activity in *GBA1* mutant neurons. Pharmacological mTOR inhibition restored TFEB activity and improved neuronal proteostasis. Additionally, inhibiting sphingolipid biosynthesis using the lipid substrate-reducing compound Genz-123346 (GZ) effectively decreased mTORC1 kinase activity and increased TFEB levels in *GBA1* mutant neurons. Our study uncovers a new mechanism contributing to proteinopathy in *GBA1-*associated PD. It also demonstrates the involvement of lipid imbalance in PD pathogenesis through the deregulation of mTORC1-TFEB axis.

## Methods

### Differentiation of iPSCs to DA neurons

**Figure 1:**
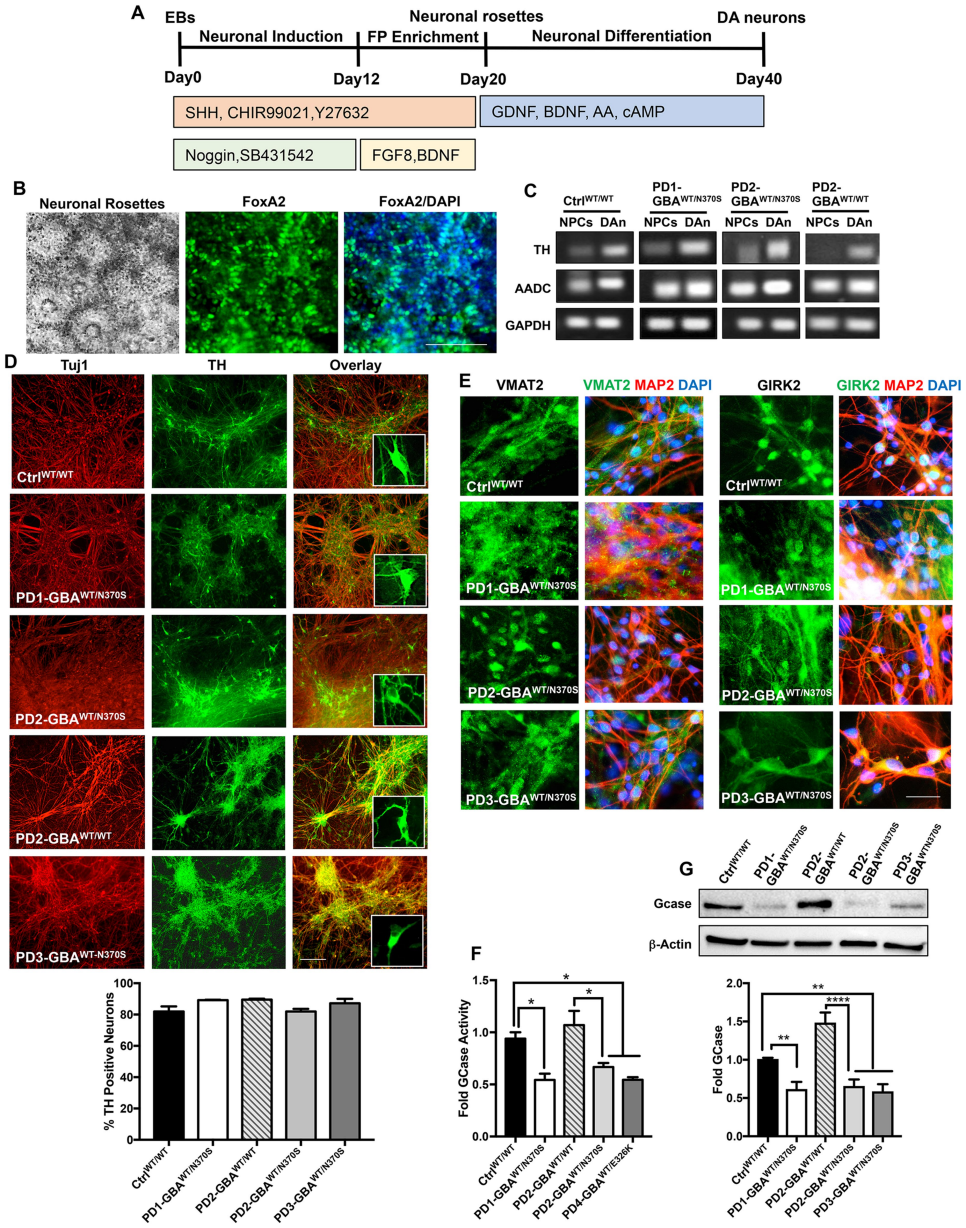
Generation of human iPSC model of *GBA1*-associated PD. **(A)** Schematic overview of the protocol used to differentiate iPSCs to dopaminergic (DA) neurons. Enrichment for midbrain floor-plate (FP) progenitors was initiated by culturing iPSCs-derived embryoid bodies (EBs) for 12 days in the presence of Noggin, SB431542 (SMAD signal inhibitors), CHIR99021 (WNT-β catenin signal activator), Sonic hedgehog (SHH) and Y27632 (ROCK inhibitor), followed by additional 8 days in the presence of FGF8 and BDNF. Neuronal rosettes appearing by day 20, were manually picked and differentiated to DA neurons by culturing in media supplemented with ascorbic acid (AA), cAMP, BDNF, and GDNF for 2–3 weeks. **(B)** Representative phase contrast image for iPSC-neuronal rosettes appearing around day 20 of the differentiation protocol showing the characteristic appearance. Also shown are immunofluorescence images of neuronal progenitors within the rosettes expressing the floor-plate marker FoxA2 and its overlay with nuclear DAPI. Scale bar = 100um. **(C)** Representative RT-PCR analysis showing expression of the dopamine synthesis enzymes; Tyrosine hydroxylase (TH), and Aromatic l-amino acid decarboxylase (AADC) in dopaminergic neurons (DAn) and neuronal progenitor cells (NPCs) differentiated from WT control (Ctrl ^WT/WT^), *GBA1* mutant, and gene-corrected PD iPSC lines. GAPDH is used as a loading control. **(D)** Representative immunofluorescence images of dopaminergic neuronal cultures (DNCs) differentiated from WT control and the indicated PD iPSC lines. Neurons were co-labeled with the pan-neuronal marker, Tuj1 (red) and the DA neuron marker, TH (green). Also shown in the last panels are the overlay of both markers and an enlargement of TH positive neurons in each line. Scale bar = 100 μm. Bar graph below shows the percentage of TH positive neurons in WT control and the indicated PD DNCs. Neurons were counted in at least 3 different fields per experiment in 2–3 independent experiments. Data represent average ± SEM. **(E)** Representative immunofluorescence images of WT control and the indicated *GBA1* mutant PD DNCs labeled with an antibody to the vesicular monoamine transporter 2 (VMAT2), or the G-protein regulated inward-rectifier potassium channel 2 (GIRK2). Also shown is the overlay of each marker (green) with MAP2 (red) and DAPI (blue). Scale bar = 50 μm. **(F)** GCase enzyme activity in WT control, *GBA1* mutant, and gene-corrected PD NPCs. Data represent average fold activity relative to control in triplicate wells in a representative experiment ± SEM. *p = 0.02 between the indicated groups as assessed by One-way ANOVA. **(G)** Western blot analysis showing GCase protein levels in WT control, *GBA1* mutant, and gene-corrected PD DNCs. Also shown is β-Actin loading control. Bar graph shows fold GCase relative to the WT control. Data represent average ± SEM. *n* = 3–4 per group. **p = 0.006 (WT vs. PD1), **p = 0.008 (WT vs. PD2), **p = 0.004 (WT vs. PD3), and ****p < 0.0001 between the indicated groups as assessed by One-way ANOVA.

In this study, we used four PD patients’ iPSC lines harboring heterozygous *GBA1* mutations (Table 1). Those lines were derived from two PD patients with *GBA1*-N370S mutations, and a third patient with *GBA1*-E326K mutation. PD1-GBA^WT/N370S^ (ID ND29756) and an age-matched control line (Ctrl^WT/WT^) (ID ND34791), were generously provided by Dr. Zeng (Buck Institute for Age Research, CA) and were maintained in culture as previously described (Momcilovic et al., 2016). The following PD iPSC lines and the CRISPR/Cas9-corrected isogenic ones were obtained from the NIH/NINDS, Human Cell and Data Repository: PD2-GBA^WT/N370S^ (ID NH50187), PD2-GBA^WT/WT^ (ID NH50186), PD3-GBA^WT/N370S^ (ID NH50183), PD4-GBA^WT/E326K^ (ID NH50143), and PD4-GBA^WT/WT^ (ID NH50142). Sequencing verification data for the gene-edited lines (NH50186 and NH50142) are provided in the Supplementary material section. Both PD2-GBA^WT/N370S^ and PD3-GBA^WT/N370S^ iPSC lines were derived from the same patient and were used interchangeably in various experiments. PD iPSCs were maintained in feeder-free conditions in Matrigel-coated plates (Corning) and mTeSR medium (Stem Cell Technologies), supplemented with Pen/Strep (1X vol/vol) and 20 ng/mL Thermostable basic FGF (Sigma-Aldrich). iPSCs were grown into embryoid bodies (EB) as previously described (Awad et al., 2017) and maintained for 4–6 days in medium supplemented with 10 μM Y27632 (R&D systems) before transferring to Matrigel-coated plates to initiate neuronal induction. DA differentiation was carried out using a previously described method with minor modifications (Hartfield et al., 2014). This protocol involves two steps of neuronal induction and enriching for midbrain floor plate progenitors, followed by manual picking of DA progenitors and differentiation into DA neurons (Figure 1A). For the first 12 days, cells where maintained in DMEM/F12 medium supplemented with 2 mM L-glutamine, 1X (volo/vol), N2 supplement (Life Technologies), 1 mg/mL Bovine Serum Albumin (Sigma-Aldrich) and penicillin/streptomycin (Life Technologies), in the presence of 10 μM Y27632, 10 μM SB431542 (Sigma-Aldrich), 200 ng/mL noggin (R&D Systems), 500 ng/mL sonic hedgehog (R&D Systems), and 0.7 μM CHIR99021 (R&D Systems). After 12 days, the same media was continued without SB431542 and noggin and with decreasing sonic hedgehog concentration (20 ng/mL). We also added 20 ng/mL Brain Derived Neurotrophic Factor (BDNF) (R&D Systems), 200 μM L-Ascorbic Acid (Sigma-Aldrich), and 100 ng/mL FGF8 (R&D Systems). Cells were maintained in this media for additional 8–10 days and until the appearance of dense neuronal rosettes containing DA progenitors, which were manually picked and cryopreserved or plated onto glass bottom dishes (Mat-Tek) coated with poly L-Ornithine hydrobromide and Laminin (both from Sigma-Aldrich) for differentiation to DA neurons. Differentiation medium consisted of Neurobasal medium (Life Technologies) supplemented with 1X (vol/vol) MEM non-essential amino acids (Life Technologies), 1X (vol/vol) GlutaMAX-I (Life Technologies), 1X (vol/vol) B27 supplement (Life Technologies), 1X (vol/vol) penicillin/streptomycin, 200 μM L-Ascorbic acid (Sigma-Aldrich), 100 nM N6,2′-O-dibutyryladenosine 3′,5′-cyclic monophosphate sodium salt (d-cAMP) (Sigma), 10 ng/mL BDNF (R&D Systems) and 10 ng/mL GDNF (R&D Systems). DA Differentiation continued for 4–6 weeks before neurons were used for various experimental procedures. For quantitation of TH expression, fluorescence microscopy images were acquired at 20× magnification from at least three independent fields. The percentage of TH expression was calculated as the number of TH-positive neurons divided by the number of DAPI-positive nuclei after excluding nuclei that are not associated with Tuj1 or MAP2 expression in the same vision field. Neuronal Progenitor cells (NPCs) were maintained in culture as previously described (Awad et al., 2015), or plated in Matrigel-coated chamber slides (Lab-Tek) for immunofluorescence analysis.

**Table 1:** iPSC lines used in the study.

iPSC Line	ID
Ctrl^WT/WT^	ND34791
PD1-GBA^WT/N370S^	ND29756
PD2-GBA^WT/N370S^	NH50187
PD2-GBA^WT/WT^	NH50186
PD3-GBA^WT/N370S^	NH50183
PD4-GBA^WT/E326K^	NH50143
PD4-GBA^WT/WT^	NH50142

### Chemical reagents and cell treatment

For mTOR inhibition, NPCs or DNCs were treated with 200 nM Torin1 (Sigma-Aldrich) for 16–18 h were indicated. For autophagic flux assay, DNCs were treated with the lysosomal inhibitor Chloroquine diphosphate (100 μm) (Invitrogen) for 18 h in the presence or absence of Torin1. For lipid substrate reduction treatment, DNCs were treated with 5 μM of the Glucosylceramide Synthase Inhibitor, Genz-123346 (Sigma-Aldrich) for 3–5 days.

### TFEB activity assay

TFEB transcriptional activity was measured using Human TFEB Activity Assay Kit (RayBiotech) according to the manufacturer’s instruction. Cell lysates were prepared in RIPA buffer (TEKnova) supplemented with Protease /Phosphotase Inhibitor (Cell Signaling), followed by sonication. Protein concentration was measured using MicroBCA Protein Assay kit (Thermo Scientific). TFEB assay was performed in duplicate wells using 30–60 μg total protein per well and measured using a plate reader.

### Reverse transcription polymerase chain reaction

mRNA was extracted using the RNeasy kit (QIAGEN). Equal RNA amounts were reverse transcribed using iScript cDNA synthesis kit (BioRad). Polymerase chain reaction (PCR) reactions were prepared using Taq 2X master mix (New England BioLabs). Sequences of all the primers used for PCR analysis are listed in Supplementary Table S1.

### GCase assay

GCase enzyme activity was measured as previously described (Panicker et al., 2012). Briefly, NPCs were incubated with the fluorescence-conjugated substrate, 4-methylumbelliferyl β-d-glucopyranoside (Sigma-Aldrich) in the presence or absence of conduritol B epoxide (CBE) (Sigma-Aldrich) to control for non-GCase enzymatic activity. GCase activity was determined in triplicate wells using a fluorescence plate reader after background subtraction.

### Immunofluorescence analysis

For immunofluorescence analysis, cells were fixed in 4% (Vol/Vol) paraformaldehyde for 15 min then blocked in 8% (Vol/Vol) HyClone Fetal Bovine Serum (Thermo Scientific) for 1 h at room temperature. This was followed by incubation with primary antibodies in blocking buffer containing 0.2% Saponin (Sigma-Aldrich) over night at 4°C. Cells were then washed with PBS and incubated with the corresponding fluorochrome-conjugated secondary antibodies (Alexa Fluor 488 or Alexa Fluor 594, Invitrogen) at 1:500 for 1 h at room temperature. Cells were mounted in Vectashield mounting medium containing DAPI (VectorLabs) to label the nuclei. The following primary antibodies were used (all at 1:200 dilution unless otherwise stated): anti-LMX1 (Millipore), anti-Nurr1 (R&D Systems), anti-VMAT2 (Proteintech, 20873-1-AP), anti-GIRK2 (Proteintech, 21647-1-AP and Millipore, AB5200), anti-MAP2 (Millipore, MAB3418), anti-FoxA2 (Millipore, AB4125), anti-mTOR (Cell Signaling, 2972) 1:100, anti-phospho-mTOR-Ser2448 (Cell Signaling, 5536) 1:100, anti-S6 Ribosomal Protein (Cell Signaling, 2217) 1: 200, anti-phosphoS6-Ser235/236 (Cell Signaling, 2211 and 4856) anti-LAMP1 (U. Iowa Developmental Hybridoma Bank, H4A3) 1:100, anti-Tuj1 (Neuromics, MO15013 and BioLegend, 801207), anti-TFEB (Bethyl Laboratories, A303-673A), anti-Tyrosine Hydroxylase (Sigma-Aldirch, T1299), and anti-Bip/Grp78 (abcam, ab21685).

### Fluorescence images acquisition and quantitation

Fluorescence images were captured using Revolve microscope with Olympus optics and 8MP color camera at 60X or 20X magnification (Echo Laboratories). High-resolution images were captured using a Zeiss LSM-510 confocal microscope (Carl Zeiss) and an AxioCam digital microscope camera at 40X magnification. For fluorescence signal quantitation, all images within each experiment were acquired at the same microscope settings. Fluorescence intensities were analyzed using ImageJ software (NIH) after background subtraction and threshold detection.

### Western blot analysis

Cells were lysed in RIPA buffer supplemented with Protease /Phosphotase Inhibitor, or directly lysed in Laemmli buffer (Bio-Rad) containing β-Mercaptoethanol (Sigma-Aldrich). Following sonication, protein lysates were denatured in loading buffer at 95°C for 5 min, loaded onto 4–20% polyacrylamide gels (Bio-Rad), and analyzed by electrophoresis. The proteins were then transferred to PVDF or nitrocellulose membranes, blocked in 5% BSA for one hour and incubated with the primary antibodies overnight at 4°C followed by incubation with the corresponding HRP-conjugated secondary antibodies (Cell Signaling, 7074P2 and 7076P2) for one hour. Anti-β-Actin-HRP conjugated antibody (Sigma-Aldrich, A3854), anti β-tubulin antibody (Cell Signaling, 5346S), or anti-GAPDH (Sigma-Aldrich, G8795) were used to control for loading. Membranes were developed using Chemiluminescent Substrates and imaged using Chemidoc imager and Imagelab software (BioRad). The following primary antibodies were used (all at 1:1000 dilution): anti-mTOR (Cell Signaling, 2983), anti-phospho-mTOR-Ser2448 (Cell Signaling, 5536), anti-S6 Ribosomal Protein (Cell Signaling, 2217), anti-phospho-S6-Ser235/236 (Cell Signaling, 4856), anti-phospho-4EBP1-Thr37/46 (Cell Signaling Cat. No. 2855), anti-4E-BP1 (Cell Signaling, 9452), anti-LAMP1, anti-p62 (BD, 610832), anti-NBR1 (Cell Signaling, 9891), anti-TFEB (MyBioSource, MBS855552) (Bethyl Laboratories, A303-673A), anti-phosphoTFEB-Ser142 (Millipore, ABE1971), anti-Phospho-α-Synuclein (Ser129) (Cell Signaling, 23706), anti-LC3A/B (Cell Signaling, 12741), anti-GBA (Sigma WH0002629M1), anti-Galectin 3 antibody (Thermo Fisher, MA1940), anti-Bip Bip/Grp78 (abcam, ab21685), anti-eIF2 (Proteintech, 66482), anti-Phospho-eIF2 (Ser 51) (ABclonal, AP0692), and anti-ATF4 (Proteintech, 10835-1-AP).

### Quantitative real-time PCR

For gene expression analysis NPCs or DNCs were cultured in 24-well plates. mRNA was extracted using the RNAeasy kit (Qiagen) and cDNA was synthesized using iScript cDNA synthesis kit (Bio-Rad). Gene expression was determined by quantitative PCR (7900 HT, Applied Biosystems) in duplicate wells using SYBR Green PCR Master Mix (Thermo Fisher). Relative gene expression was normalized to the corresponding value of GAPDH expression in each sample and the fold changes relative to the control values within the same experiment were determined using the 2^–∆∆Ct^ method. The sequence of all the primers used for qRT-PCR analysis are listed in Supplementary Table S2.

### Statistical analysis

Data were analyzed using One-way ANOVA followed by Tukey’s or Sidak’s post-test to determine statistical differences between multiple groups. Two-tailed unpaired Student’s t-tests were used for comparison between two groups when appropriate. In some figures, data were combined from DNCs generated from multiple PD iPSC lines (all with *GBA1*-N370 mutations) as indicated in the figure legends. *p* values <0.05 were considered statistically significant. The confidence level for significance was 95%. Data were analyzed using Prism software version 7.0a (GraphPad Software, La Jolla, CA).

## Results

### Generation of iPSC model of *GBA1*-associated PD

In this study, we used four PD patients’ iPSC lines harboring heterozygous *GBA1* mutations. One line was derived from a patient with *GBA1*-N370*S* mutation (PD1-GBA^WT/N370S^). Two lines were from another patient also with *GBA1*-N370S mutation (PD2-GBA^WT/N370S^ and PD3-GBA^WT/N370S^). The fourth line was from a patient with the *GBA1*-E326K mutation (PD4-GBA^WT/E326K^). Both the N370S and E326K are common *GBA1* mutations in PD patients (Do et al., 2019; Avenali et al., 2020). PD1-GBA^WT/N370S^ and an age-matched wild-type (WT) control line (Ctrl^WT/WT^) were generously provided by Dr. Zeng (Buck Institute for Research on Aging, CA) and were previously published (Momcilovic et al., 2016). All the other PD iPSC lines and the CRISPR/Cas9-corrected isogenic control lines (PD2-GBA^WT/WT^ and PD4-GBA^WT/WT^), were obtained from the NINDS/NIH, Human Cell and Data Repository. Table 1 lists all the iPSC lines used in this study and the corresponding *GBA1* mutation in each. Both control and PD iPSCs were differentiated into midbrain DA neurons by following a previously reported method with minor modifications (Hartfield et al., 2014). Briefly, iPSC-embryoid bodies were subjected to neuroectodermal induction and enrichment for midbrain floor progenitors according to the protocol outlined in Figure 1A. Neuronal rosettes appearing around day 20 showed a high expression of the floor-plate marker, FoxA2 (Figure 1B), indicating successful enrichment of DA progenitors. Neuronal rosettes were manually picked and differentiated into DA neurons or maintained in culture as neuronal progenitor cells (NPCs). Immunofluorescence analysis showed a high expression of the DA progenitor markers FoxA2 and Nurr1 in both control and PD NPCs (Supplementary Figure S1A). NPCs also expressed LMX1, indicating their midbrain identity (Supplementary Figure S1B). RT-PCR analysis showed a marked induction in the expression of the dopamine synthesis enzymes, Tyrosine hydroxylase (TH) and Aromatic l-amino acid decarboxylase (AADC) in dopaminergic neuronal cultures (DNCs) as compared to the parent NPCs (Figure 1C; Supplementary Figure S1C), indicating successful enrichment of DA neurons. Quantitation of the percentage of TH-positive neurons within the pan-neuronal markers (Tuj1 or MAP2)- positive population indicated that approximately 80% of the neurons were TH-positive and that both control and PD iPSC lines exhibited a similar differentiation efficiency (Figure 1D; Supplementary Figure S1D). In addition, both control and PD DNCs showed a similar expression of the mature DA neuron markers, the G-protein-regulated inward-rectifier potassium channel 2 (GIRK2), and the vesicular monoamine transporter 2 (VMAT2) (Figure 1E; Supplementary Figure S1E). To further characterize our PD iPSC model, we compared GCase enzyme activity in control and *GBA1* mutant NPCs. Our data showed approximately 50% reduction in GCase activity in PD NPCs harboring *GBA1* mutations as compared to WT control cells, which is consistent with the effect of the heterozygous mutations (Figure 1F). On the other hand, GCase activity in the PD gene-corrected NPCs was similar to the WT control and significantly higher than the parent cells, indicating successful correction of the *GBA1* mutations (Figure 1F; Supplementary Figure S2A). Western blot (WB) analysis confirmed the significant reduction in GCase levels in PD DNCs and its restoration in the isogenic gene-corrected cells (Figure 1G). Thus, we have successfully generated neuronal cultures enriched for DA neurons from PD patients’ iPSC lines, which retain the effects of *GBA1* mutations on GCase level and activity. We also confirmed the successful correction of the *GBA1* mutations in the gene-edited cells.

### Decreased TFEB activity in *GBA1* mutant PD neurons

**Figure 2:**
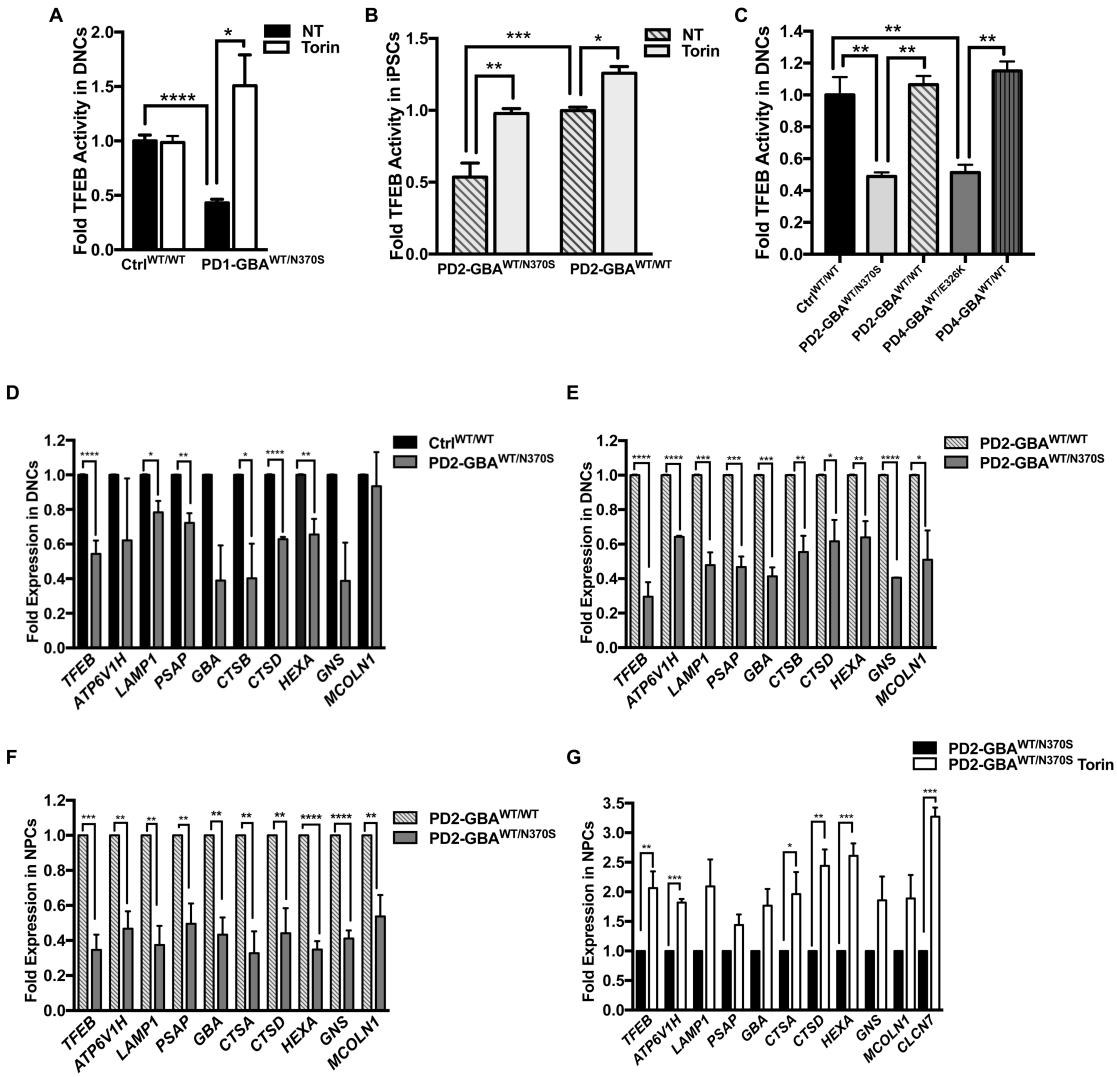
Decreased TFEB activity in *GBA1* mutant PD neurons. Measurement of TFEB activity in: **(A)** WT control and *GBA1* mutant PD DNCs. *p = 0.04 and ***p = 0.0003 between the indicated groups as assessed by Student’s *t*-test. **(B)** *GBA1* mutant PD iPSCs and the corresponding isogenic gene-corrected line. *p = 0.03, **p = 0.001, and ***p = 0.0002 between the indicated groups as assessed by One-way ANOVA. **(C)** WT control, *GBA1* mutant, and gene-corrected PD DNCs. **p = 0.009 (WT vs. PD2), **p = 0.006 (PD2 vs. PD2 gene-corrected), **p = 0.005 (WT vs. PD4), and **p = 0.001 (PD4 vs. PD4 gene-corrected) as assessed by One-way ANOVA. Cells were either untreated or treated with 200 nM Torin1 for 18 h where indicated. Data represent average fold activity relative to control ± SEM, *n* = 3–4 per group. **(D)** qRT-PCR analysis showing expression of selected TFEB target genes in WT control and *GBA1* mutant PD DNCs. Data represent fold relative to control ±SEM, *n* = 3–4 per group. ****p < 0.0001 (TFEB), *p = 0.01 (LAMP), *p = 0.04 (CTSB), ****p<0.0001 (CTSD), ***p = 0.0006 (HEXA), and **p = 0.001 (PSAP), as assessed by Student’s *t*-test. **(E)** qRT-PCR analysis showing expression of TFEB target genes in *GBA1* mutant and gene-corrected PD DNCs. Data represent fold relative to gene-corrected cells ±SEM in 2–3 independent experiments. *p = 0.03 (MCOLN), *p = 0.02 (CTSD), **p = 0.001 (HEXA), **p = 0.009 (CTSB), ***p = 0.0001 (PSAP), ***p = 0.0004 (LAMP,GBA), and ****p < 0.0001 (TFEB, ATP6, GNS) as assessed by Student’s *t*-test. **(F)** qRT-PCR analysis showing fold expression of TFEB target genes in *GBA1* mutant and gene-corrected PD NPCs. Data represent fold relative to gene-corrected cells ±SEM in 3–4 independent experiments. **p = 0.001 (ATP6,CTSA),**p = 0.007 (CTSD), **p = 0.009 (MCOLN), **p = 0.004 (LAMP,PSAP),**p = 0.001 (GBA),***p = 0.0003 (TFEB), and ****p < 0.0001 (HEXA, GNS) as assessed by Student’s *t*-test. **(G)** qRT-PCR analysis showing expression of TFEB target genes in *GBA1* mutant NPCs that were untreated or treated for 18 h with 200 nM Torin1. Data represent fold relative to untreated cells ±SEM in 2–3 independent experiments. *p = 0.04, **p = 0.002 (CTSD), **p = 0.009 (TFEB), ***p = 0.0003 (CLCN7), and ***p = 0.0002 (ATP6, HEXA) as assessed by Student’s *t*-test.

We have previously demonstrated altered TFEB-mediated lysosomal biogenesis in neuropathic GD neurons harboring homozygous *GBA1* mutations (Awad et al., 2015). To investigate the involvement of TFEB dysfunction in *GBA1*-associated PD, we conducted a TFEB activity assay using whole-cell lysates extracted from control and *GBA1* mutant PD neurons. This ELISA-based assay detects active (non-phospho) TFEB in cellular lysate and measures its binding to the CLEAR motif. Our data showed a significant reduction in TFEB activity in PD DNCs harboring *GBA1-*N370S mutation as compared to the WT control cells (Figure 2A). Treatment with Torin1, a pharmacological mTOR kinase inhibitor and a known TFEB activator (Martina et al., 2012), successfully increased TFEB activity in the mutant neurons but did not cause a significant change in control cells (Figure 2A). To determine whether TFEB alterations are due to the effect of *GBA1* mutation, we compared TFEB activity in the CRISPER/Cas9-corrected iPSCs and DNCs to the parent *GBA1* mutant cells. We detected approximately a 50% increase in TFEB activity in PD cells corrected for the *GBA1-*N370S mutation compared to the parent mutant cells (Figures 2B,C) and treatment with Torin1 increased the activity in both the mutant and the gene-edited iPSCs (Figure 2B). To test if this effect is specific to the *GBA1-*N370S mutation, we compared TFEB activity in PD DNCs harboring the *GBA1-*E326K mutation, which is known to predispose to PD but not cause GD (Duran et al., 2013). Again, we found approximately 50% decrease in TFEB activity in *GBA1-*E326K mutant DNCs compared to both the WT control and the isogenic-corrected cells (Figure 2C). On the other hand, there was no significant difference in TFEB activity between PD DNCs that are corrected for either *GBA1-*N370S or *GBA1-*E326K mutations compared to the WT control neurons, indicating that correcting *GBA1* mutations successfully restored TFEB activity (Figure 2C). Thus, our data demonstrate that heterozygous *GBA1* mutations result in decreased TFEB activity, which can be restored by pharmacological mTOR inhibition.

### Attenuation of the CLEAR network gene expression in *GBA1* mutant PD neurons

Next, we examined the ALP gene expression in PD DNCs, which is dependent on TFEB activity (Settembre and Medina, 2015). qRT-PCR analysis for a set of TFEB target genes containing the CLEAR motif in their promotors showed a significant decrease in the expression levels of many of these genes in DNCs harboring *GBA1-*N370S mutations compared to WT control cells (Figure 2D). ALP genes that were examined included: *ATP6V1H, LAMP1, GBA, cathepsins (CATS) D,B* and *A, HEXA, GBA1, MCOLN1, GNS*, and *PSAP*. These genes encode integral lysosomal proteins that are important for the ALP functions (Martini-Stoica et al., 2016). Consistent with TFEB regulation of its own expression (Settembre et al., 2013), we also detected a significant decrease in *TFEB* levels in PD mutant DNCs compared to control cells (Figure 2D). Additionally, the expression levels of *TFEB* and its target genes were significantly reduced in both *GBA1* mutant DNCs (Figure 2E) and NPCs (Figure 2F), compared to the corresponding isogenic gene-corrected cells. On the other hand, there was no significant decrease in TFEB target gene expression between WT control and gene-corrected DNCs (Supplementary Figure S2B), indicating that attenuation of the CLEAR network gene expression is due to the effect of *GBA1* mutations. Our TFEB assay data indicated that Torin1 successfully restored TFEB activity in *GBA1* mutant cells. We tested whether TFEB activation using Torin1 would also restore TFEB target gene expression. qRT-PCR analysis showed a significant increase in the expression levels of *TFEB* and its target genes in the PD NPCs treated with Torin1 compared to untreated cells (Figure 2G). Together our data indicate that *GBA1* mutations diminish TFEB transcriptional activity, thus causing a subsequent attenuation of the CLEAR network gene expression in PD neurons. It also shows that pharmacological mTOR inhibition successfully upregulates TFEB activity in those cells.

### Decreased TFEB levels in *GBA1* mutant PD neurons

**Figure 3:**
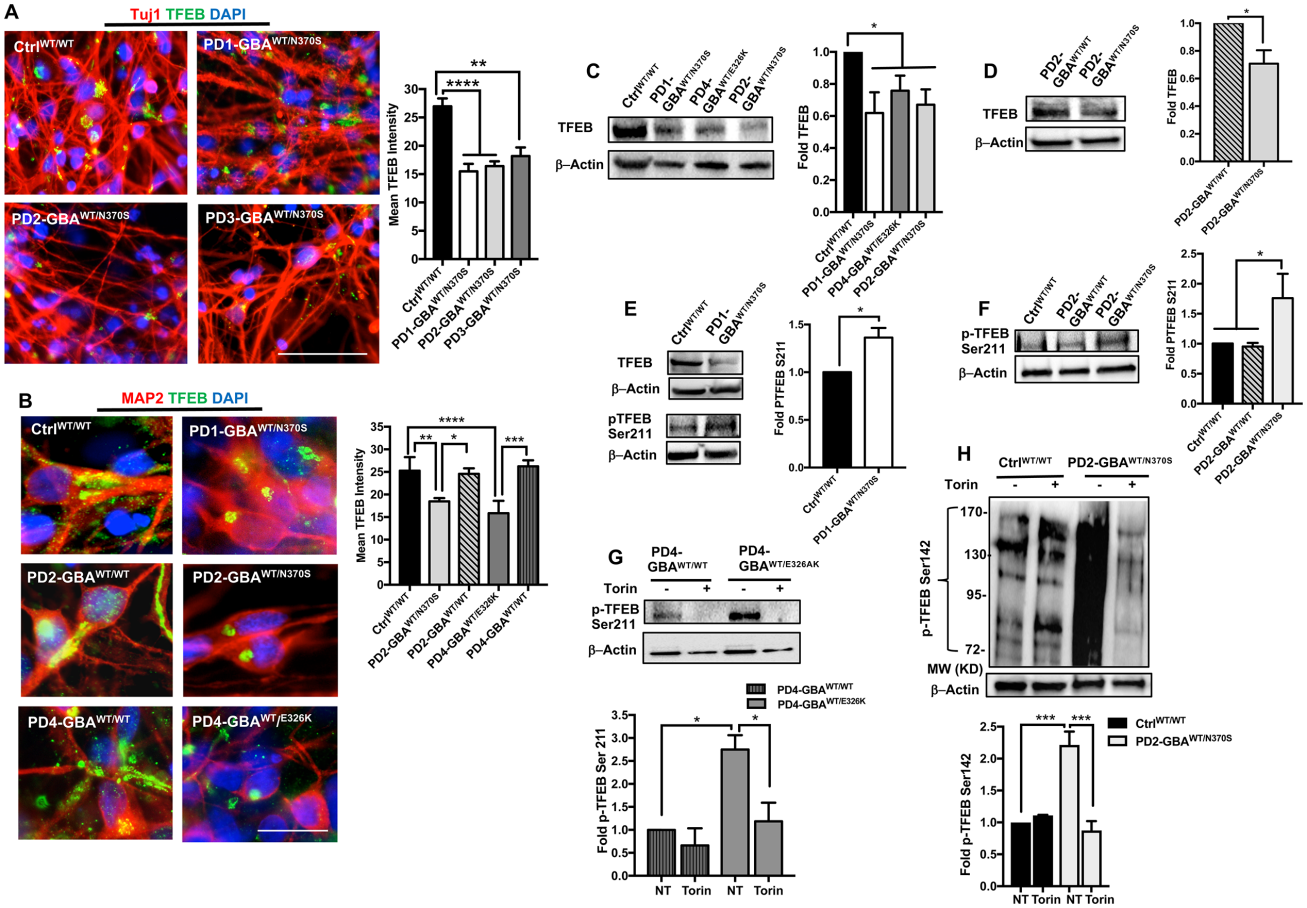
Decreased TFEB levels in *GBA1* mutant PD neurons. **(A)** Representative immunofluorescence images for WT control and *GBA1* mutant PD neurons co-labeled with anti-Tuj1 (red) and anti-TFEB (green) antibodies. Also shown is nuclear DAPI (blue), (images with separate fluorescence channels are provided in Supplementary Figure S3A). Scale bar = 50 μm. Bar graph represents quantitation of TFEB fluorescence signal intensity. Compiled data from >200 cells per group assayed in at least 3 different fields per experiment in 2–5 independent experiments. Error bars = SEM. **p = 0.002 and ****p < 0.0001 between the indicated groups as assessed by One-way ANOVA. **(B)** Representative immunofluorescence images for WT control, *GBA1* mutant, and gene-corrected neurons co-labeled with anti-MAP2 (red) and anti-TFEB (green) antibodies. Also shown is nuclear DAPI (blue). Scale bar = 25 μm. Bar graph represents quantitation of TFEB fluorescence signal intensity. Compiled data from >200 cells per group assayed in at least 3 different fields per experiment in 2–5 independent experiments. Error bars = SEM. *p = 0.01, **p = 0.001, ***p = 0.0002, and ****p < 0.0001 between the indicated groups as assessed by One-way ANOVA. **(C)** Western blot analysis for TFEB levels in WT control and *GBA1* mutant PD DNCs. Also shown is β-Actin loading control. Bar graph represents fold TFEB relative to control, Data represent average ± SEM, *n* = 3 per group. *p = 0.01 (WT vs. PD4), *p = 0.02 (WT vs. PD2), and *p = 0.04 (WT vs. PD1) as assessed by Student’s *t*-test. **(D)** Western blot analysis for TFEB levels in *GBA1* mutant and gene-corrected PD DNCs. Also shown is β-Actin loading control. Bar graph represents fold TFEB relative to the gene-corrected cells. Data represent average (PD2-GBA^WT/N370S^ and PD3-GBA^WT/N370S^ combined) ± SEM, *n* = 4 per group. *p = 0.02 as assessed by Student’s *t*-test. **(E)** Western blot analysis for total TFEB and pTFEB (Ser211) levels in control and *GBA1* mutant PD DNCs. Also shown is β-Actin loading control. Bar graph represents fold pTFEB (Ser211) relative to control. Data represent average ± SEM, *n* = 2–3 per group. *p = 0.01 as assessed by Student’s *t*-test. **(F)** Western blot analysis for pTFEB (Ser211) levels in WT control, *GBA1* mutant, and gene-corrected PD DNCs. Also shown is β-Actin loading control. Bar graph represents fold pTFEB (Ser211) relative to control. Data represent average ± SEM, *n* = 2–4 per group. *p = 0.03 (WT vs. mutant) and *p = 0.02 (mutant vs. gene-corrected) as assessed by One-way ANOVA. **(G)** Western blot analysis for pTFEB (Ser211) levels in *GBA1* mutant and gene-corrected PD DNCs. Also shown is β-Actin loading control. Cells were either untreated or treated with 200 nM Torin1 for 18 h. Bar graph represents fold pTFEB (Ser211) relative to gene-corrected cells. Data represent average ± SEM, *n* = 2–3 per group. *p = 0.01 between the indicated groups as assessed by One-way ANOVA. **(H)** Western blot analysis for p-TEFB (Ser142) levels in control and *GBA1* mutant PD DNCs. Also shown is β-Actin loading control. Cells were either untreated or treated with 200 nM Torin1 for 18 h. Bar graph represents fold pTFEB (Ser142) relative to untreated control. Data represent average ± SEM, *n* = 3 per group. ***p = 0.0007 (WT vs. mutant), and ***p = 0.0003 (mutant vs. mutant with Torin) as assessed by One-way ANOVA.

Our qRT-PCR data showed decreased *TFEB* gene expression in *GBA1* mutant PD DNCs. Next, we examined whether TFEB protein levels are also reduced in the mutant cells. Immunofluorescence (IF) analysis showed a significant reduction in TFEB fluorescence signal intensity in PD neurons compared to both WT control and the isogenic gene-corrected cells (Figures 3A,B; Supplementary Figure S3A). We also noticed that in WT control neurons, TFEB fluorescence signal was strong and spread throughout the cytoplasm, but in PD neurons, the signal was weak and mainly localized to a small area in the supranuclear region (Figure 3B). Interestingly, gene-correcting the *GBA1* mutations restored both TFEB fluorescence signal intensity and expression pattern in the gene-edited PD neurons (Figure 3B). WB analysis confirmed the significant decrease in TFEB levels in PD DNCs harboring *GBA1* mutations compared to both WT control (Figure 3C), and the isogenic-corrected neurons (Figure 3D; Supplementary Figure S3B). To further characterize TFEB alterations in *GBA1* mutant PD neurons, we examined its phosphorylation status. It is known that TFEB is phosphorylated by mTORC1 on two key Serine residues, Ser211 and/or Ser142, which occurs in response to nutrient-rich conditions and modulates its nuclear translocation and activity (Martina et al., 2012; Settembre et al., 2012; Napolitano et al., 2018). Using a phospho-specific antibody, we compared p-TFEB (Ser211) levels between control and PD neurons. Our data showed a significant increase in p-TFEB (Ser211) levels in PD DNCs compared to both WT control and the corresponding gene-corrected cells (Figures 3E–G). Importantly, there was no significant difference in p-TFEB (Ser211) levels between WT control and the gene-edited neurons, confirming that increased TFEB phosphorylation is mediated by the *GBA1* mutation (Figure 3F). To test the effect of inhibiting mTOR activity on TFEB phosphorylation status, we treated PD DNCs with Torin1 and measured p-TFEB (Ser211) levels. As expected, Torin1 reduced p-TFEB (Ser211) levels in both *GBA1* mutant DNCs and the corresponding gene-edited cells, however, the inhibitory effect was only significant in the mutant DNCs compared to untreated cells (Figure 3G). Next, we examined TFEB phosphorylation on Ser142, another important modification that targets inactive TFEB for ubiquitination and proteasomal degradation (Sha et al., 2017). We previously showed increased p-TFEB (Ser142) levels and decreased TFEB stability in GD iPSC neurons (Brown et al., 2019). Consistent with our previous results, WB analysis of p-TFEB (Ser142) showed a dark-smeared band of high molecular weight in PD DNCs harboring *GBA1*-N370S mutations but not in control cells, suggesting excess TFEB ubiquitination in the mutant cells (Figure 3H). However, we did not obtain similar results on increased p-TFEB (Ser142) levels in PD DNCs with *GBA1*-E326K (data not shown), suggesting that (Ser211) might be the primary mTORC1 phosphorylation site of TFEB in those PD neurons. Similar to p-TFEB (Ser211), treatment with Torin1 effectively decreased p-TFEB (Ser142) levels in *GBA1* mutant PD neurons to control levels (Figure 3H). Thus, our data indicate that *GBA1* mutations result in decreased levels and increased mTOR-dependent phosphorylation of TFEB in PD neurons.

### Decreased TFEB nuclear translocation in *GBA1* mutant PD neurons

**Figure 4:**
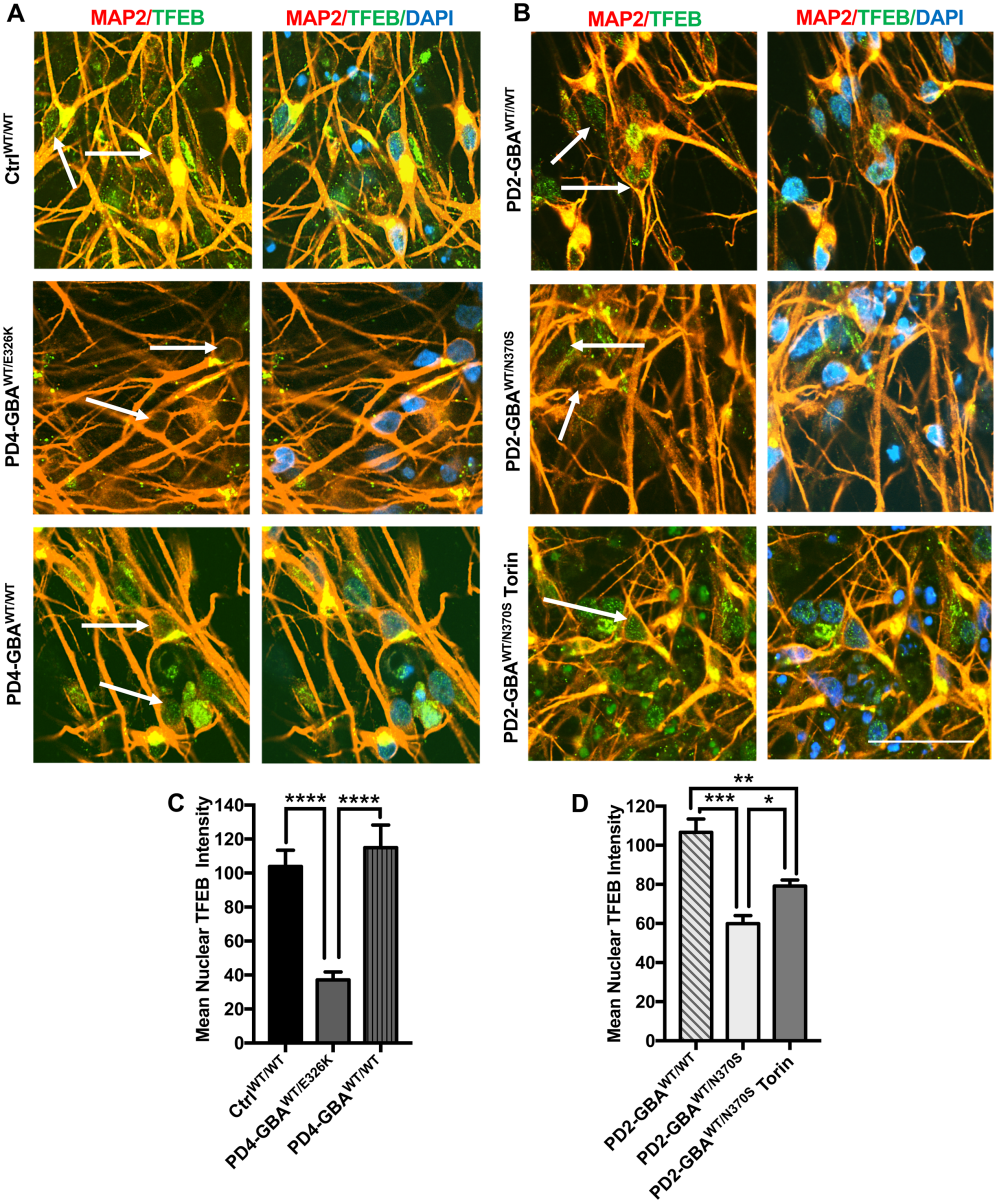
Decreased nuclear TFEB levels in *GBA1* mutant PD neurons. **(A)** Representative immunofluorescence images for WT control, *GBA1* mutant, and gene-corrected PD neurons co-labeled with anti-MAP2 (red) and anti-TFEB (green) antibodies. Also shown is nuclear DAPI (blue). Arrows point to nuclear TFEB fluorescence signal, (images with separate fluorescence channels are provided in Supplementary Figure S4). Scale bar = 25um. **(B)** Representative immunofluorescence images for gene-corrected and *GBA1* mutant neurons that was untreated or treated with 200 nM Torin1 for 18 h. Cells were co-labeled with anti-MAP2 (red) and anti-TFEB (green) antibodies. Also shown is nuclear DAPI (blue). Arrows point to nuclear TFEB signal. **(C)** Quantitation of nuclear TFEB fluorescence signal intensity in WT control, *GBA1* mutant, and gene-corrected neurons. Data were collected from >50 cells per group, assayed in 3 different fields in a representative experiment. Data represent average ± SEM. ****p < 0.0001 between the indicated groups as assessed by One-way ANOVA. **(D)** Quantitation of nuclear TFEB fluorescence signal intensity in gene-corrected, *GBA1* mutant, and Torin-treated *GBA1* mutant neurons. Data were collected from >50 cells per group, assayed in 3 different fields in a representative experiment. Data represent average ± SEM. *p = 0.04, **p = 0.008, and ***p = 0.0001 between the indicated groups as assessed by One-way ANOVA.

It is known that TFEB phosphorylation on Ser211 and/or Ser142 inhibits its activity by preventing its nuclear translocation (Napolitano and Ballabio, 2016; Napolitano et al., 2018). Our data showed increased TFEB phosphorylation on both Serine residues in *GBA1* mutant DNCs. To investigate the effect of increased TFEB phosphorylation on its cellular localization, we acquired high- resolution confocal images and examined TFEB fluorescence signal co-localization with nuclear DAPI. We notice that, in addition to the decreased overall TFEB fluorescence intensity in PD neurons, TFEB expression in the nuclear compartment was also reduced (Figures 4A,B; Supplementary Figure S4A). On the other hand, the reduction in nuclear TFEB signal was not observed in the gene-corrected PD neurons (Figures 4A,B; Supplementary Figure S4A). Quantitation of TFEB fluorescence signal intensity in the nuclear compartment confirmed the significant reduction of its levels in the mutant neurons compared to WT control cells (Figure 4C). It also showed that nuclear TFEB expression in the gene-corrected neurons is significantly higher than the parent mutant neurons and similar to WT control levels (Figures 4C,D). These results indicate that correcting *GBA1* mutations successfully restored TFEB’s ability to translocate to the nuclei of PD neurons. Our data showed that pharmacological mTOR inhibition increases TFEB transcriptional activity in the *GBA1* mutant cells. We asked whether this effect is mediated by the enhancement of TFEB nuclear translocation. Fluorescence signal quantitation indicated a partial, but yet significant restoration of nuclear TFEB expression in the mutant neurons in response to Torin1 treatment (Figure 4D; Supplementary Figure S4B). These results indicate that *GBA1* mutations decrease TFEB nuclear translocation in PD neurons, which can be partially restored by pharmacological mTOR inhibition.

### Increased mTORC1 activity in *GBA1* mutant PD neurons

**Figure 5:**
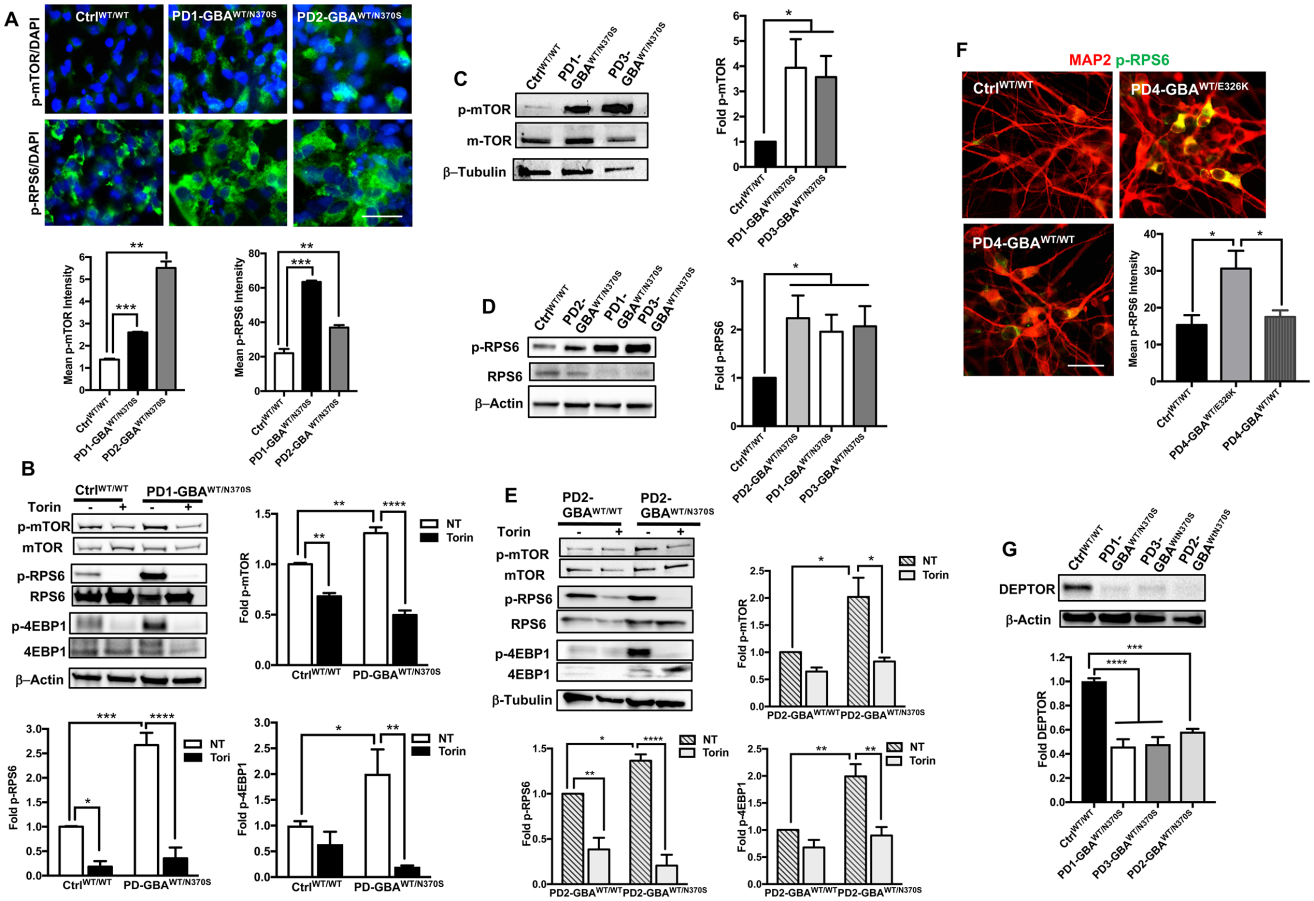
Increased mTORC1 activity in *GBA1* mutant PD neurons. **(A)** Representative immunofluorescence images of WT control and *GBA1* mutant NPCs labeled with antibodies to phospho-mTOR-Ser2448 (p-mTOR) or phospho-RPS6 Ser235/236 (p-RPS6). Also shown is nuclear DAPI. Scale bar = 25 μm. Bar graphs below represent p-mTOR or p-RPS6 fluorescence signal intensity. Data were collected from >30 cells per group, assayed in 3 different fields in a representative experiment. Data represent average ± SEM, **p = 0.001 and ***p = 0.0002 between WT control and indicated groups as assessed by Student’s *t*-test. **(B)** Western blot analysis for p-mTOR, p-RPS6 and phospho-4EBP1 Thr37/46 (p-4EBP1) levels in WT control and *GBA1* mutant PD NPCs. Also shown is β-Actin loading control. Cells were either untreated or treated with 200 nM Torin1 for 18 h. Bar graphs represent folds relative to untreated control cells. Data represent average ± SEM. *n* = 2–3 per group. For p-mTOR, **p = 0.008 (WT vs. WT with Torin), **p = 0.005 (WT vs. mutant), and ****p < 0.0001 (mutant vs. mutant with Torin). For p-RS6, *p = 0.04 (WT vs. WT with Torin), ***p = 0.0007 (WT vs. mutant), and ****p < 0.0001 (mutant vs. mutant with Torin). For p-4EBP1, *p = 0.04 (WT vs. mutant), and **p < 0.005 (mutant vs. mutant with Torin), as assessed by One-way ANOVA. **(C)** Western blot analysis for p-mTOR levels in WT control and *GBA1* mutant PD DNCs. Also shown is β-Tubulin loading control. Bar graph represents folds relative to control cells. Data represent average ± SEM, *n* = 3 per group. *p = 0.02 (WT vs. PD1) and *p < 0.01 (WT vs. PD3) as assessed by Student’s *t*-test. **(D)** Western blot analysis for p-RPS6 levels in WT control and *GBA1* mutant PD DNCs. Also shown is β-Actin loading control. Bar graph represents fold p-RPS6 relative to WT control. Data represent average ± SEM, *n* = 3 per group. *p = 0.02 between the control and indicated groups as assessed by Student’s *t*-test. **(E)** Western blot analysis for p-mTOR, p-RPS6 and p-4EBP1 levels in gene-corrected and *GBA1* mutant PD DNCs. Cells were either untreated or treated with 200 nM Torin1 for 18 h. Also shown is β-Tubulin loading control. Bar graphs represent folds relative to untreated, gene-corrected cells. Data represent average ± SEM, *n* = 3–5 per group. For p-mTOR, *p = 0.02 (gene-corrected vs. mutant), and *p < 0.01 (mutant vs. mutant with Torin). For p-RS6, **p = 0.002 (gene-corrected vs. gene-corrected with Torin), *p = 0.03 (gene-corrected vs. mutant), and ****p < 0.0001 (mutant vs. mutant with Torin). For p-4EBP1, **p = 0.003 (gene-corrected vs. mutant), and **p < 0.001 (mutant vs. mutant with Torin) as assessed by One-way ANOVA. **(F)** Representative immunofluorescence images of WT control, *GBA1* mutant and gene-corrected neurons co-labeled with antibodies to p-RPS6 (green) and MAP2 (red). Scale bar = 50 μm. Bar graph represents p-RPS6 fluorescence signal intensity. Data were collected from >25 cells per group assayed in 2 different fields, in a representative experiment. Data represent average ± SEM, *p = 0.01 (WT vs. mutant) and *p = 0.03 (mutant vs. gene-corrected) as assessed by One-way ANOVA. **(G)** Western blot analysis for DEPTOR levels in WT control and *GBA1* mutant PD DNCs. Also shown is β-Actin loading control. Bar graph represents fold relative to WT control. Data represent average ± SEM, *n* = 3–4 per group. ****p < 0.0001 and ***p = 0.0002 between the indicated groups as assessed by One-way ANOVA.

mTORC1-mediated phosphorylation of TFEB is known to inhibit its nuclear translocation and target inactive TFEB for proteasomal degradation (Settembre and Medina, 2015; Sha et al., 2017). Our data showed a significant reduction in both TFEB levels and activity in *GBA1* mutant DNCs. To investigate whether TFEB alterations are mediated by the deregulation of the upstream mTOR signal, we examined mTORC1 kinase activity in *GBA1* mutant NPCs and DNCs. IF analysis showed a marked increase in the levels of mTOR phosphorylated at Ser2448 (active p-mTOR) in PD NPCs as shown by increased fluorescence signal intensity (Figure 5A). We also found a significant increase in the fluorescence intensity of the phosphorylated ribosomal protein S6 (RPS6) in PD NPCs compared to control cells (Figure 5A). RPS6 is a known downstream target of mTORC1 and its phosphorylation status reflects mTORC1 kinase activity (Ikenoue et al., 2009). On the other hand, fluorescence signal intensity of total mTOR and RPS6 were not significantly different between PD NPCs and WT control cells (Supplementary Figure S5). WB analysis confirmed the significant increase in the levels of p-mTOR and its downstream effectors; p-RPS6 and the phosphorylated eukaryotic initiation factor 4E-binding protein 1 (4EBP1) in PD NPCs as compared to control cells (Figure 5B). Treatment with Torin1 effectively reduced the levels of p-mTOR and its effectors; p-RPS6 and p-4EBP1 in both control and PD NPCs (Figure 5B). Next, we compared mTORC1 kinase activity in DNCs differentiated form control and *GBA1* mutant NPCs. Again, we found a significant increase in the levels of p-mTOR and its downstream effector p-RPS6 in PD DNCs harboring *GBA1-*N370S mutations as compared to control cells (Figures 5C,D). To determine if the increased mTORC1 activity is linked to the effect of *GBA1* mutation, we compared mTORC1 activity in PD DNCs to the isogenic gene-corrected cells. We found a significant increase in the levels of p-mTOR and its downstream effectors, p-RPS6 and p-4EBP1 in PD DNCs harboring *GBA1-*N370S mutations as compared to the gene-corrected cells (Figure 5E). We also found a significant increase in p-RPS6 levels in *GBA1-* E326K mutant neurons compared to both the WT control and the corresponding gene-corrected cells (Figure 5F). Furthermore, we found a significant decrease in the levels of the DEP domain-containing mTOR interacting protein (DEPTOR) in PD DNCs as compared to both the WT control (Figure 5G) and the corresponding gene-corrected cells (Supplementary Figure S6A). It is known that DEPTOR is an important endogenous inhibitor of mTORC1 kinase activity (Walchli et al., 2021) and a reduction in its levels indicate diminished negative regulation of mTORC1 in the *GBA1* mutant neurons.

### ALP dysfunction in *GBA1* mutant PD neurons is mediated by increased mTOR activity

**Figure 6:**
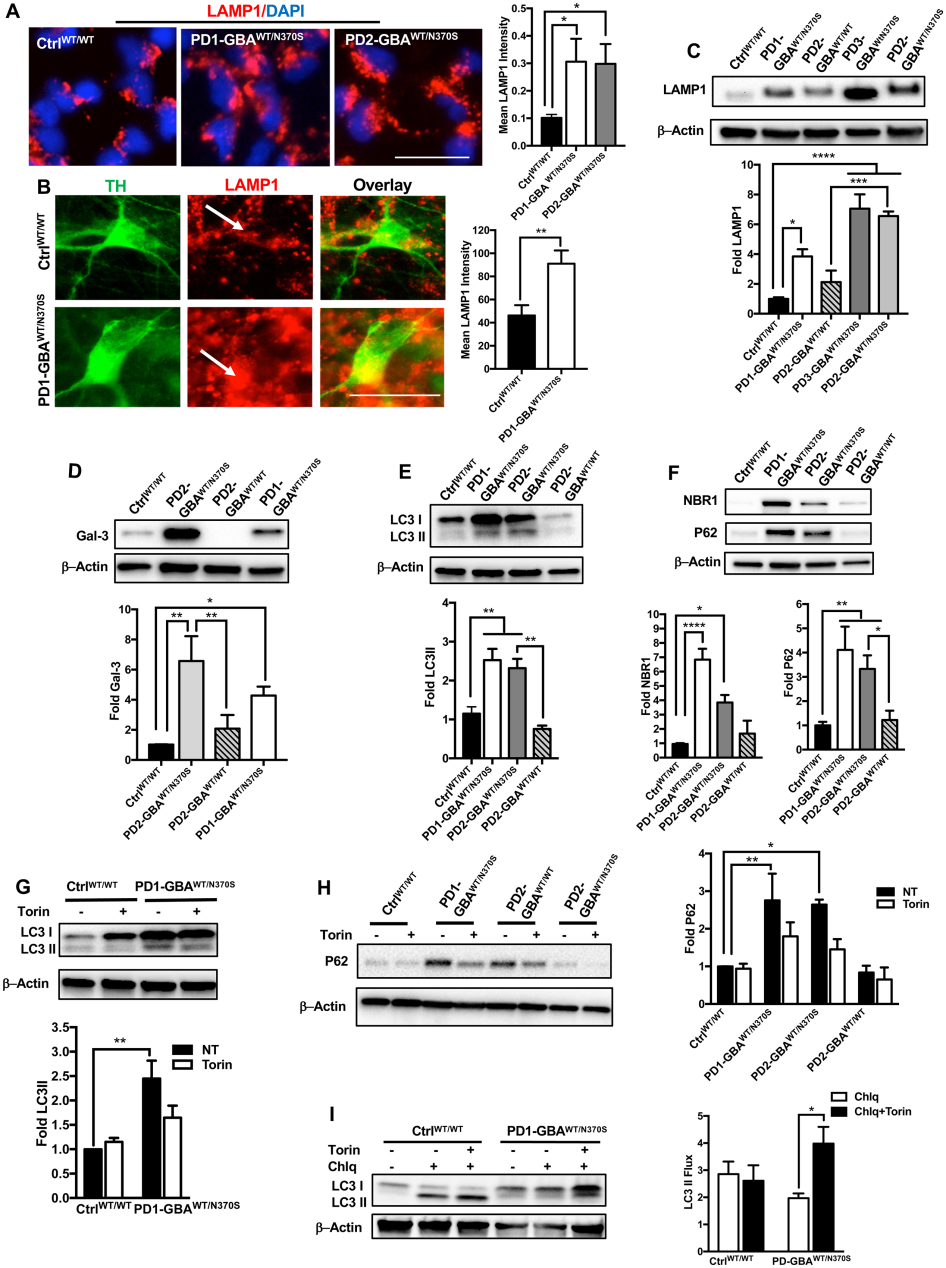
mTOR inhibition improves autophagic clearance in *GBA1* mutant PD neurons. **(A)** Representative immunofluorescence images of WT control and *GBA1* mutant NPCs labeled with an antibody to LAMP1. Also shown is nuclear DAPI. Scale bar = 25 μm. Bar graph represent mean LAMP1 fluorescence signal intensity. Data were collected from >30 cells per group, assayed in 3 different fields in a representative experiment. Data represent average ± SEM, *p = 0.04 (WT vs. PD1) and *p = 0.02 (WT vs. PD2) as assessed by One-way ANOVA. **(B)** Representative immunofluorescence images of WT control and *GBA1* mutant neurons co-labeled with anti-LAMP1 and anti-TH. Last panel is the overlay of the two markers. Arrows point to LAMP1-labeled puncti in TH expressing neurons. Scale bar = 25 μm. Bar graph represent mean LAMP1 fluorescence signal intensity in TH positive neurons. Data were collected from >30 cells per group, assayed in 2–3 different fields in 3 independent experiments. Data represent average ± SEM, **p = 0.007 between the indicated groups as assessed by Student’s *t*-test. **(C)** Western blot analysis for LAMP1 levels in WT control, *GBA1* mutant, and gene-corrected PD DNCs. Also shown is β-Actin loading control. Bar graph represents fold LAMP1 relative to control, Data represent average ± SEM, *n* = 3–4 per group. *p = 0.01, ***p = 0.0002 and ****p < 0.0001 between the indicated groups as assessed by One-way ANOVA. **(D)** Western blot analysis for Galectin3 (Gal3) levels in WT control, *GBA1* mutant, and gene-corrected PD DNCs. Also shown is β-Actin loading control. Bar graph represents fold Gal3 relative to control. Data represent average ± SEM, *n* = 3–4 per group. *p = 0.01, **p = 0.001 (WT vs. PD2), and **p = 0.004 (PD2 vs. gene-corrected) as assessed by One-way ANOVA. **(E)** Western blot analysis for LC3II levels in WT control, *GBA1* mutant, and gene-corrected PD DNCs. Also shown is β-Actin loading control. Bar graph represents fold LC3II relative to control. Data represent average ± SEM, *n* = 3–4 per group. **p = 0.005 (WT vs. PD1), **p = 0.009 (WT vs. PD2), and **p = 0.002 (PD2 vs. gene-corrected) as assessed by One-way ANOVA. **(F)** Western blot analysis for NBR1 and P62 levels in WT control, *GBA1* mutant, and gene-corrected PD DNCs. Also shown is β-Actin loading control. Bar graph represents folds relative to control. Data represent average ± SEM, *n* = 3–6 per group. For NBR1, ****p<0.000 (WT vs. PD1) and *p=0.02 (WT vs. PD2). For P62, *p = 0.03 (PD2 vs. gene-corrected), **p = 0.009 and (WT vs. PD2), **p = 0.003 and (WT vs. PD1) as assessed by One-way ANOVA. **(G)** Western blot analysis for LC3II levels in WT control and *GBA1* mutant PD DNCs. Also shown is β-Actin loading control. Cells were either untreated or treated with 200 nM Torin1 for 18 h. Bar graph represents fold LC3II relative to untreated control. Data represent average ± SEM, *n* = 3–4 per group. **p = 0.002 between the indicated groups as assessed by One-way ANOVA. **(H)** Western blot analysis for P62 levels in WT control, *GBA1* mutant, and gene-corrected PD DNCs. Also shown is β-Actin loading control. Cells were either untreated or treated with 200 nM Torin1 for 18 h. Bar graph represents folds relative to untreated control. Data represent average ± SEM, *n* = 3–4 per group. **p = 0.008 (WT vs. PD1) and *p = 0.01 (WT vs. PD2) as assessed by One-way ANOVA. **(I)** Western blot analysis for autophagic flux in WT control, and *GBA1* mutant PD DNCs. Cells was either untreated or treated with Torin1 or Torin1 plus Chloroquine (chlq) for18 h as indicated. Bar graph represents quantification of LC3 flux normalized to actin. Data represent average (from PD1-GBA^WT/N370S^ and PD2-GBA^WT/N370S^ combined) ± SEM, *n* = 3–6 per group. *p = 0.02 between the indicated groups as assessed by One-way ANOVA.

Our data indicate negative regulation of TFEB by mTORC1 in *GBA1* mutant PD neurons and NPCs. TFEB is the master regulator of the ALP (Napolitano and Ballabio, 2016), thus we thought to examine the ALP functions in those mutant cells. First, to visualize the lysosomal compartment, we labeled control and mutant NPCs with an antibody against the lysosomal-associated membrane protein 1 (LAMP1) and examined LAMP1-labeled puncta, which corresponds with lysosomal abundance. We found a significant increase in LAMP1 expression in PD NPCs compared to control cells indicating an expanded lysosomal compartment in the mutant cells (Figure 6A). Consistent with previous reports (Schondorf et al., 2014), increased LAMP1 expression was also detected in DNCs harboring heterozygous *GBA1* mutations as shown by both IF (Figure 6B) and WB analysis (Figure 6C). On the other hand, in the gene-corrected PD DNCs, LAMP1 levels were significantly less than the parent DNCs and similar to control levels, indicating that increased LAMP1 expression is mediated by *GBA1* mutations (Figure 6C). Since reduced TFEB activity is expected to decrease lysosomal biogenesis, we reasoned that increased LAMP1-labeled puncta in PD neurons may indicate damaged or defective lysosomes rather than newly formed ones. In support of this idea, it has been shown that LAMP1 labels a heterogenous population of the endolysosomal compartment, which also includes lysosomes that are lacking degradative capacities (Cheng et al., 2018). To test this possibility, we examined the expression of Galectin-3 (Gal3), a β-galactoside-binding cytosolic lectin that is used as a reporter for endolysosomal damage (Jia et al., 2020). As anticipated, we found a significant increase in Gal3 levels in PD DNCs as compared to both WT control and gene-corrected cells, indicating accumulation of damaged lysosomes in the mutant cells (Figure 6D). We next examined lysosomal clearance of autophagic substrates, which is dependent on TFEB activity. WB analysis of the autophagosome marker; Microtubule-associated protein 1A/light chain 3 (LC3), indicated a significant increase in LC3II levels in PD DNCs compared to WT control and gene-corrected cells (Figure 6E). LC3II is known to associate with the autophagosomal membranes and an increase in its levels reflects autophagic perturbation in the mutant neurons (Klionsky et al., 2012). To further assess autophagic clearance, we compared levels of p62/SQSTM1 (p62) and NBR1 in control and PD mutant neurons. Both are scaffolding proteins that target ubiquitinated substrates for autophagic degradation (Kirkin et al., 2009). Our data showed a significant increase in both p62 and NBR1 levels in PD DNCs compared to both WT control and gene-corrected cells, indicating defective lysosomal clearance of autophagic substrates in the mutant cells (Figure 6F). We then tested whether mTOR inhibition (which increases TFEB activity) would also improve lysosomal clearance in PD DNCs. As anticipated, treatment with Torin 1 reduced both LC3II and p62 levels in PD DNCs indicating efficient lysosomal clearance of autophagic substrates in response to treatment (Figures 6G,H). To further characterize the effect of mTOR inhibition on the autophagic defect in PD DNCs, we conducted a flux assay in the presence and absence of Torin1. DNCs were treated with a lysosomal inhibitor (Chloroquine) to block autophagosomal degradation and examined LC3-II levels, which under these conditions will reflect autophagosome formation independent from the clearance process (Ueno and Komatsu, 2020). Our data showed a significant increase in LC3II levels in PD DNCs (but not control cells) treated with Torin1 as compared to untreated cells, indicating enhancement of autophagic flux in response to treatment (Figure 6I). Together, our data indicate that autophagic perturbation in *GBA1* mutant PD neurons is linked to the increased mTOR kinase activity and can be reversed by pharmacological mTOR inhibition.

### Pharmacological mTOR inhibition decreases ER stress and reduces toxic α-synuclein accumulation in *GBA1* mutant PD neurons

**Figure 7:**
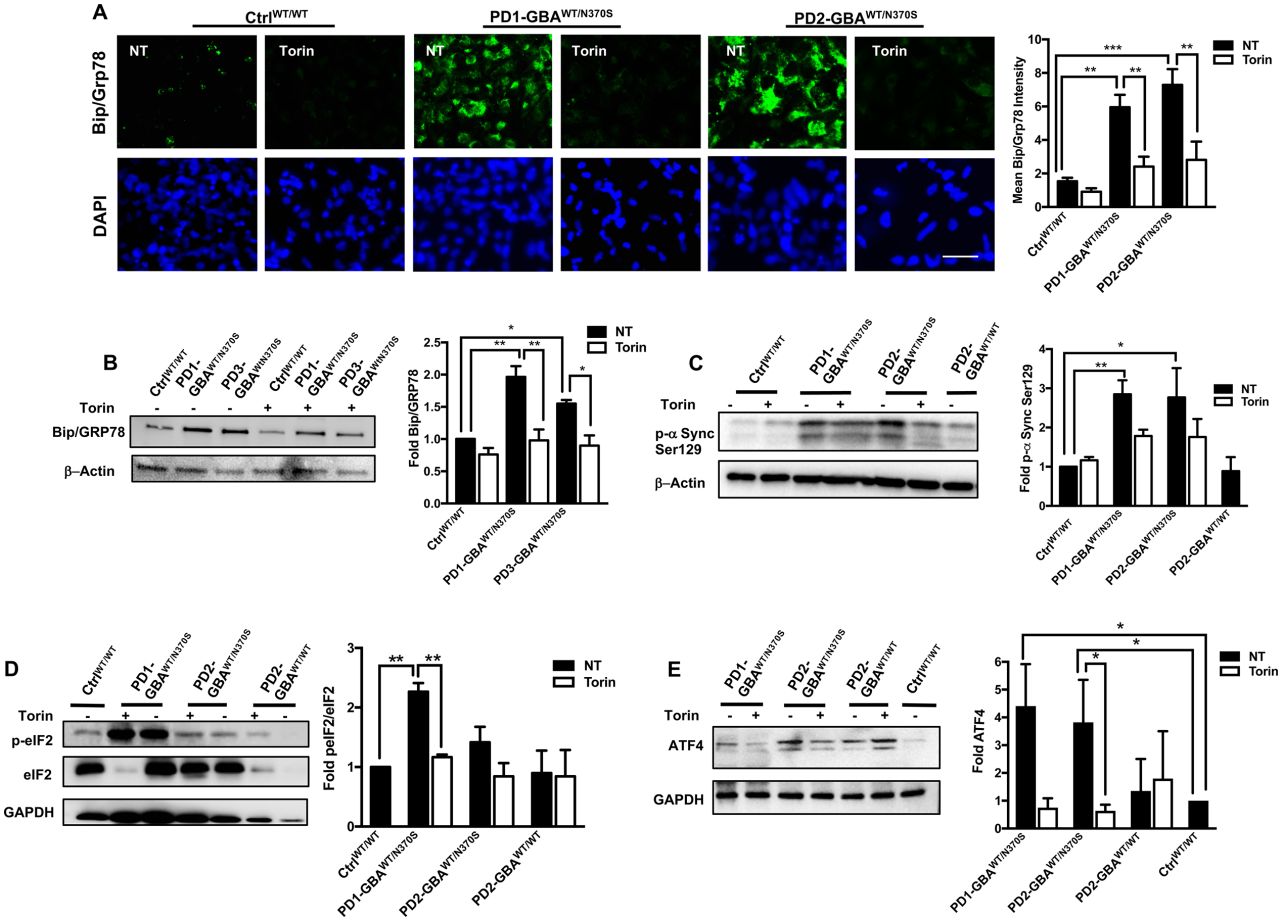
mTOR inhibition decreases ER stress and reduces toxic α-Synuclein accumulation in *GBA1* mutant PD neurons. **(A)** Representative immunofluorescence images of WT control and *GBA1* mutant NPCs labeled with an antibody to Bip/Grp78. Also shown is nuclear DAPI. Cells were either untreated (NT) or treated with 200 nM Torin1 for 18 h. Scale bar = 25 μm. Bar graph represents mean Bip/Grp78 fluorescence signal intensity. Data were collected from >30 cells per group, assayed in 3 different fields in a representative experiment. Data represent average ± SEM, **p = 0.004 (WT vs. PD1), ***p = 0.006 (WT vs. PD2), **p = 0.002 (PD1 vs. PD1 with Torin), and **p = 0.008 (PD2 vs. PD2 with Torin) as assessed by One-way ANOVA. **(B)** Western blot analysis for Bip/Grp78 levels in WT control and *GBA1* mutant PD DNCs. Also shown is β-Actin loading control. Cells were either untreated or treated with 200 nM Torin1 for 18 h. Bar graph represents folds relative to untreated control. Data represent average ± SEM, *n* = 2–3 per group. **p = 0.001 (WT vs. PD1), *p = 0.02 (WT vs. PD3), **p = 0.001 (PD1 vs. PD1 with Torin), and *p = 0.01 (PD3 vs. PD3 with Torin) as assessed by One-way ANOVA. **(C)** Western blot analysis for p-α-Synuclein (Ser 129) levels in WT control, *GBA1* mutant, and gene-corrected PD DNCs. Also shown is β-Actin loading control. Cells were either untreated or treated with 200 nM Torin1 for 18 h. Bar graph represents p-α-Synuclein (Ser 129) fold relative to untreated control. Data represent average ± SEM, *n* = 3–4 per group. *p = 0.01 and **p = 0.008 between the indicated groups as assessed by One-way ANOVA. **(D)** Western blot analysis for p-eIF2-α levels in WT control and *GBA1* mutant PD DNCs that were either untreated or treated with 200 nM Torin1 for 18 hours. Also shown is GAPDH loading control. Bar graph represents fold p-eIF2-α relative to total eIF2-α. Data represent average ± SEM, *n* = 3 per group. **p = 0.001 (WT vs. PD1) and **p = 0.007 (PD1 vs. PD1with Torin) as assessed by One-way ANOVA. **(E)** Western blot analysis for ATF4 levels in WT control, *GBA1* mutant and gene-corrected PD DNCs. Also shown is GAPDH loading control. Cells were either untreated or treated with 200 nM Torin1 for 18 hours. Bar graph represents folds relative to untreated control. Data represent average ± SEM, *n* = 2–4 per group. *p = 0.04 (WT vs. PD1), *p = 0.03 (WT vs. PD2), and *p = 0.03 (PD2 vs. PD2 with Torin) as assessed by One-way ANOVA.

*GBA1* mutations are known to cause ER stress in cellular and animal models of PD (Fernandes et al., 2016; Navarro-Romero et al., 2022). Increased ER stress results in upregulation of the unfolded protein response (UPR), which if persisted can lead to the activation of apoptotic signals (Banerjee et al., 2011; Hetz, 2012). TFEB prevents ER stress by enhancing protein clearance *via* the ALP (Martina et al., 2016). Our data showed decreased TFEB activity and autophagic perturbation in PD DNCs, which can be reversed by pharmacological mTOR inhibition. To test whether mTOR inhibition would also alleviate ER stress in PD neurons, we compared levels of Bip/GRp78 in Torin1-treated and untreated cells. Bip/GRp78 is an ER-resident chaperone that is induced under stress conditions (Park et al., 2017). Our data showed a significant increase in Bip/GRp78 protein levels in PD NPCs (Figure 7A) and DNCs (Figure 7B) harboring *GBA1* mutations, indicating increased basal ER stress levels in the mutant cells. Treatment with Torin1 significantly decreased Bip/GRp78 levels in PD cells to control levels, indicating improvement of proteostasis in response to treatment (Figures 7A,B). We next tested the effect of pharmacological mTOR inhibition on the accumulation of toxic α-synuclein, which is closely linked to PD pathology and known to promote ER stress (Wang et al., 2012; Kawahata et al., 2022). WB analysis showed a significant increase in the levels of α-synuclein phosphorylated at Ser129 (toxic α-Synuclein) in PD DNCs but not in the gene-corrected cells (Figure 7C), which is consistent with previous reports on the effect of *GBA1* mutations (Gegg et al., 2020; Glajch et al., 2021). Interestingly, treatment with Torin1, efficiently reduced p-α-Synuclein Ser129 levels in *GBA1* mutant DNCs as reflected by the absence of a significant difference in its levels between treated and WT control DNCs (Figure 7C). However, the reduction in p-α-Synuclein Ser129 levels was not statistically significant between the treated and untreated cells, suggesting that a prolonged treatment duration might be needed or other pathways need to be targeted simultaneously to achieve a stronger effect. On the other hand, Torin1 did not cause a significant change on p-α-Synuclein Ser129 levels in the gene-corrected neurons (Supplementary Figure S6B). Next, we asked whether reducing ER stress by Torin1 would also prevent UPR – mediated activation of apoptotic signals in PD neurons. To test this, we examined phosphorylation of the eukaryotic initiation factor 2 α-subunit (eIF2α), a known inducer of apoptotic gene expression downstream of the Protein kinase RNA-like endoplasmic reticulum (PERK) kinase (Rozpedek et al., 2016). Our data showed an increase in the levels of p- eIF2α and its downstream effector; the activating transcription factor 4 (ATF4) in PD DNCs indicating upregulation of the UPR in the mutant neurons (Figures 7D,E). Treatment with Torin1 reduced eIF2α phosphorylation and restored AFT4 to control levels (Figures 7D,E). Interestingly, we did not detect a significant increase in ATF4 in the gene-corrected PD DNCs compared to control WT cells, confirming that the UPR upregulation is due to the effect of *GBA1* mutations (Figure 7E). Together these data indicate that pharmacological mTOR inhibition successfully improves proteostasis in *GBA1* mutant PD neurons.

### Treatment with lipid-substrate reducing compound decreases mTORC1 activity and increases TFEB expression in *GBA1* mutant PD neurons

**Figure 8:**
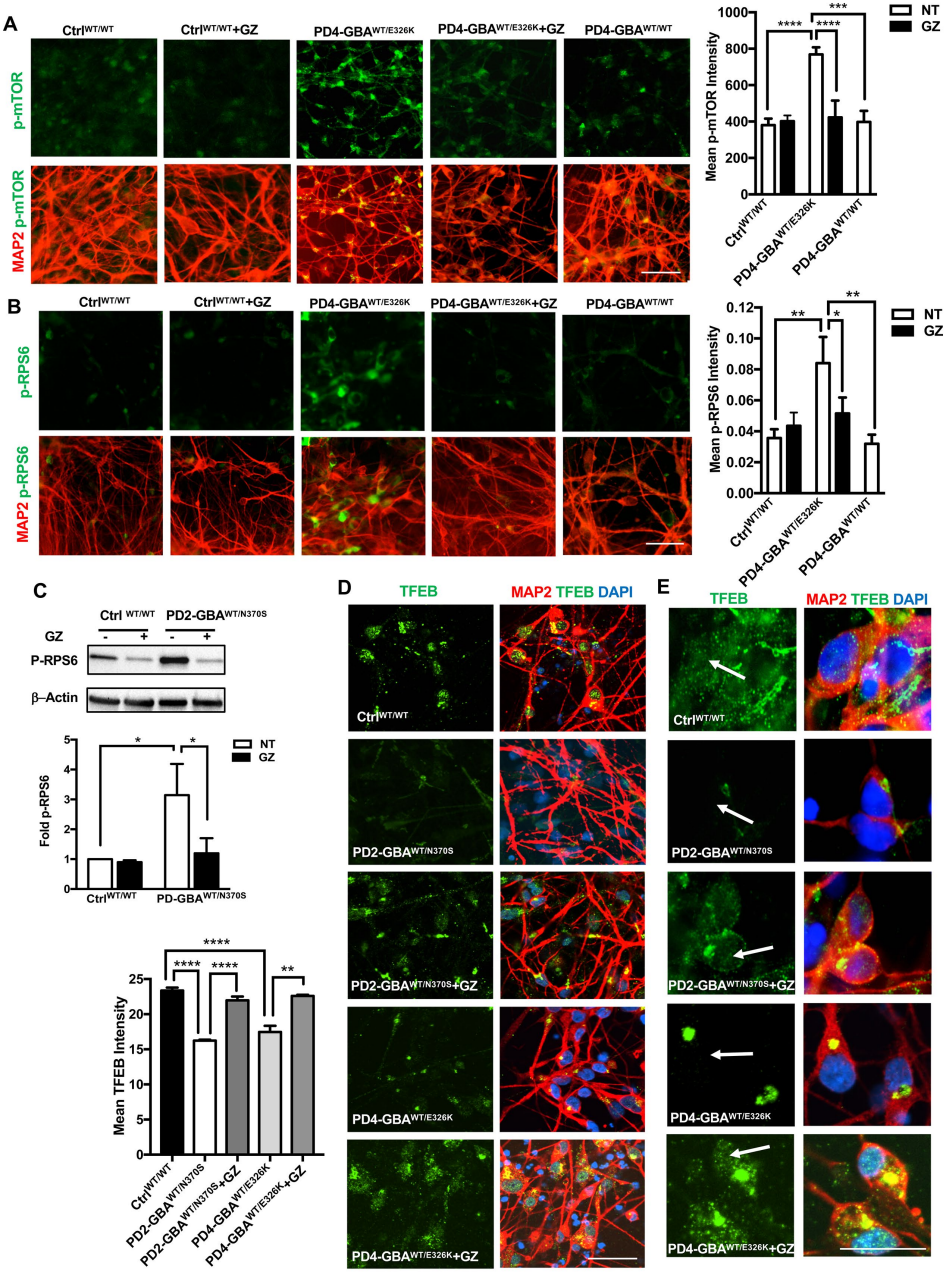
Treatment with Glucosylceramide synthase inhibitor decreases mTORC1 activity and increases TFEB expression in *GBA1* mutant PD neurons. **(A)** Representative immunofluorescence images of WT control, gene-corrected, and *GBA1* mutant neurons co-labeled with anti-p-mTOR (green) and anti-MAP2 (red). Also shown is nuclear DAPI. *GBA1* mutant neurons were either untreated or treated with 5 μM of the lipid substrate reduction compound Genz-123346 (GZ) for 3 days. Scale bar = 50 μm. Bar graph represents mean p-mTOR fluorescence signal intensity ±SEM. Data were collected at least 50 cells per group, assayed in 3–4 different fields in 1–2 independent experiments. ****p<0.0001 (WT vs. PD4), ***p = 0.0004 (PD4 vs. gene-corrected), and ****p < 0.0001 (PD4 vs. PD4 GZ) as assessed by One-way ANOVA. **(B)** Representative immunofluorescence images of WT control and *GBA1* mutant neurons co-labeled with antibodies to p-RPS6 (green) and MAP2 (red). Cells were either untreated or treated with GZ. Scale bar = 50 μm. Bar graph represent mean p-RPS6 fluorescence signal intensity. Data were collected from >100 cells per group, assayed in 3 different fields per experiment in 3 independent experiments. Error bars = SEM, *p = 0.01 and **p = 0.006 between the indicated groups as assessed by One-way ANOVA. **(C)** Western blot analysis for p-RPS6 levels in WT control and *GBA1* mutant PD DNCs. Also shown is β-Actin loading control. Cells were either untreated or treated with 5 μM GZ for 3–5 days. Bar graph represents folds relative to untreated control cells. Data represent average (from PD1-GBA^WT/N370S^, PD2-GBA^WT/N370S^ and PD3-GBA^WT/N370S^ combined) ± SEM, *n* = 3–5 per group. *p = 0.01 (between WT and PD) and *p = 0.02 (between PD and PD with GZ) as assessed by One-way ANOVA. **(D)** Representative immunofluorescence images of WT control and *GBA1* mutant neurons co-labeled with antibodies to TFEB (green) and MAP2 (red). Also shown is nuclear DAPI. *GBA1* mutant neurons were either untreated or treated with 5 μM GZ for 3 days. Scale bar = 25 μm. Bar graph to the left represents mean TFEB fluorescence signal intensity. Data were collected from >50 cells per group, assayed in 3 different fields in 2–3 independent experiments. Error bars = SEM, ****p < 0.0001 and **p = 0.006 between the indicated groups as assessed by One-way ANOVA. **(E)** Representative immunofluorescence images of WT control and *GBA1* mutant neurons co-labeled with antibodies to TFEB (green) and MAP2 (red). Also shown is nuclear DAPI. *GBA1* mutant neurons were either untreated or treated with 5 μM GZ for 3 days. Arrows point to TFEB fluorescence signal colocalization with nuclear DAPI. Scale bar = 50 μm.

Decreased GCase enzyme activity results in the accumulation of its lipid substrates, GlcCer and GlcSph (Do et al., 2019). We previously demonstrated that increased mTORC1 activity in GD neurons is linked to lipid substrate accumulation (Brown et al., 2019). Previous studies reported increased glycosphingolipid levels in PD DA neurons harboring heterozygous *GBA1* mutations and in PD patients’ brains (Schondorf et al., 2014; Huebecker et al., 2019). To test whether lipid substrate accumulation is involved in mTORC1 hyperactivity in PD neurons, we treated PD DNCs with the glucosylceramide synthase inhibitor Genz-123346 (GZ). This compound inhibits the activity of the glucosylceramide synthase enzyme, which promotes the synthesis of GlcCer, thus preventing its accumulation (Gupta et al., 2010). We previously demonstrated that pharmacological glucosylceramide synthase inhibition effectively decreases levels of both GlcCer and GlcSph in GD neuronal cells (Srikanth et al., 2021). Our results showed that GZ treatment significantly reduced p-mTOR fluorescence intensity in PD neurons to levels comparable to the control and the isogeneic gene-corrected cell (Figure 8A). The significant reduction in mTORC1 kinase activity in PD neurons was also confirmed by the efficient reduction of p-RPS6 to control levels in response to treatment (Figures 8B,C). Finally, we asked whether reducing mTORC1 activity using GZ would also restore TFEB expression in PD neurons. Immunofluorescence analysis indicated that GZ treatment significantly increased TFEB fluorescence signal intensity (Figures 8D,E), and enhanced TFEB signal co-localization with the nuclei in PD DNCs (Figure 8E). Together our results suggest that *GBA1*- mediated lipid substrate accumulation increases mTORC1 kinase activity, which downregulates TFEB levels and activity in PD patients’ neurons.

## Discussion

In this study, we used PD patients’ iPSC model to investigate the effect of heterozygous *GBA1* mutations on the transcriptional regulation of the autophagy-lysosomal pathway by TFEB and its involvement in PD pathology. Although *GBA1* mutations are the most common genetic risk factors for PD, the mechanisms underlying disease susceptibility remain unclear (Do et al., 2019; Behl et al., 2021). Studies using PD experimental models and patients’ samples concluded that diminished GCase enzymatic function results in two main pathogenic consequences that are likely major contributors to PD development. First, is impairing lysosomal efficiency to dispose of pathological α-synuclein, thus favoring its toxic accumulation (Mazzulli et al., 2016; Kuo et al., 2022). Second, is the glycosphingolipid substrate accumulation and subsequent alterations in cellular lipid homeostasis (Avenali et al., 2020; Navarro-Romero et al., 2022). Our study indicates that these two major pathogenic events might be mechanistically linked through deregulation of the mTORC1-TFEB axis. We found that in PD DA neurons, *GBA1*-mediated lipid substrate accumulation results in increased activity of mTORC1, the main upstream negative regulator of TFEB (Settembre et al., 2012). Subsequent reduction of TFEB activity contributes to ALP dysfunction, which favors α-synuclein accumulation.

TFEB has recently emerged as a key regulator of neuronal proteostasis by controlling the coordinated expression of the ALP genes (Song et al., 2013; Lehtonen et al., 2019). Therefore, TFEB dysfunction has been implicated in the pathogenesis of many neurodegenerative disorders including PD (Decressac et al., 2013; Cortes and La Spada, 2019). Although alterations in both mTOR and TFEB expression have been previously reported in postmortem midbrains from PD patients (Wills et al., 2012; Decressac et al., 2013; Lan et al., 2017), the contribution of this pathway to PD pathogenesis could not be investigated in this model. Our study is the first to report TFEB dysfunction in PD patients’ iPSC neurons harboring *GBA1* mutations and demonstrate its involvement in PD pathology. Studies using iPSC models of *GBA1*-associated PD reported autophagic perturbation, increased ER stress levels, and α-synuclein accumulation indicative of defective proteostasis in the mutant neurons (Schondorf et al., 2014; Fernandes et al., 2016). We detected similar alterations in our PD iPSC-DA neurons and further showed that pharmacological mTOR inhibition restores TFEB activity and enhances autophagyic clearance, thus reducing both ER stress levels and toxic α-synuclein accumulation. These results point to the involvement of *GBA1-* mediated deregulation of mTORC1-TFEB activity in promoting neuronal proteinopathy in PD and the potential therapeutic effects of targeting this pathway. Importantly, we demonstrated the absence of mTORC1-TFEB alterations and proteinopathy manifestations in the isogenic gene-corrected PD DA neurons, indicating that both defects are mediated by the *GBA1* mutations and are linked to PD pathology. Our results are in agreement with previous studies showing TFEB activation successfully ameliorates PD phenotypes in various experimental models (Sardiello, 2016). For example, in a rat model of α-synuclein toxicity, TFEB overexpression enhanced α-synuclein clearance and offered robust neuroprotection (Decressac et al., 2013). Similarly, TFEB overexpression or activation by pharmacological mTOR inhibition alleviated 6-OHDA/AA toxicity and enhanced neuronal survival in both human and mouse models of PD (Zhuang et al., 2020). Moreover, pharmacological activation of TFEB reduced cell death in MPP-treated cells and reduced α-synuclein aggregates in human neuroglioma cells (Dehay et al., 2010; Kilpatrick et al., 2015). Finally, mTOR inhibition by rapamycin is shown to reduce α-synuclein pathology both *in vitro* and *in vivo* (Martini-Stoica et al., 2016). Our results further support that restoring endogenous TFEB activity could be a promising therapeutic approach to ameliorate neurodegenerative changes in PD.

We previously identified TFEB as a critical *GBA1* target that mediates ALP dysfunction in neuropathic GD neurons (Awad et al., 2015; Brown et al., 2019). Results from this study further support the role of TFEB as a potential disease modifier in *GBA1*-associated neurodegeneration and demonstrate its involvement in PD pathogenesis. Our use of DA neurons from patients with the heterozygous *GBA1*-N370S mutation (one of the most relevant *GBA1* mutations in PD) makes our results applicable to PD patients (Do et al., 2019; Avenali et al., 2020). We also confirmed mTORC1-TFEB pathway alterations in an additional PD iPSC line harboring the *GBA1*-E326K mutation, which has been recently identified as the most prevalent PD-associated *GBA1* mutation and is known to be absent in the GD population (Duran et al., 2013; Avenali et al., 2020). The similarity of our results from both genotypes suggests that *GBA1*-mediated deregulation of the mTORC1-TFEB axis is mediated by diminished GCase enzymatic functions rather than a specific mutation-dependent effect. Furthermore, our data reflect the dose-dependent effects of the homozygous versus heterozygous *GBA1* mutations on TFEB regulation and activity. In neuropathic GD iPSC neurons, which exhibit very low GCase activity (approximately 5%), we previously reported a marked reduction in TEFB levels and subsequent alterations in lysosomal biogenesis and autophagic clearance (Awad et al., 2015; Brown et al., 2019). In this study, we detected a 50% reduction in GCase activity in PD DA neurons, which is consistent with the effect of heterozygous *GBA1* mutations. Interestingly, TFEB transcriptional activity was also decreased by a similar percentage, and gene-correcting the *GBA1* mutations successfully restored it to control levels, suggesting the dependence of TFEB activity on GCase functions.

Although diminished TFEB activity is expected to decrease lysosomal biogenesis, we and others have found an increased number of LAMP1-labeled puncta in *GBA1* mutant neurons, indicative of expanded endolysosomal compartment (Schondorf et al., 2014; Garcia-Sanz et al., 2017). This finding can be explained by previous studies showing that a significant portion of LAMP1-labeled organelles lacks major lysosomal hydrolases and thus may include defective lysosomes (Cheng et al., 2018). This is further supported by our finding that *GBA1* mutant DA neurons exhibited defective clearance of autophagic substrates and high expression of Galectin3, a known marker for damaged lysosomes (Jia et al., 2020). Similarly, a reduction in the activity of GBA and multiple other lysosomal enzymes has been reported in the substantia nigra of postmortem PD patients (Huebecker et al., 2019), which points to a potential common mechanistic basis.

We previously demonstrated mTORC1 hyperactivity in neuropathic GD neurons (Brown et al., 2019). Follow-up studies reported increased activity of the mTORC1 pathway in brain samples from both neuropathic GD patients and mice (Peng et al., 2021). Our current results indicate that *GBA1* mutations even in heterozygosity lead to increased mTORC1 kinase activity and this effect is likely mediated by the lipid substrate accumulation. In PD DA neurons, we detected decreased levels of DEPTOR, the main endogenous negative regulator of mTORC1 (Walchli et al., 2021), indicating its involvement in the cellular lipid-sensing process. Emerging evidence indicates that mTORC1 controls lipogenesis and plays a central role in modulating lipid metabolism (Lamming and Sabatini, 2013; Ricoult and Manning, 2013). Therefore, it is expected that *GBA1*-mediated lipid imbalance may trigger a feedback mechanism, leading to increased mTORC1 activity. In support of this idea, studies showed that different classes of lipids can cause direct mTORC1 activation in several animal and cellular models (Rivas et al., 2009; Menon et al., 2017; Davis et al., 2021). We and others previously reported GlcCer accumulation in iPSC neurons harboring either homozygous or heterozygous *GBA1* mutations (Panicker et al., 2012; Schondorf et al., 2014). More recently, Huebecker et al. (2019) reported a significant reduction in GBA and accumulation of its substrates, GlcCer and GlcSph in the substantia nigra of postmortem PD patients, which further highlights the pathogenic role of sphingolipid accumulation in PD pathology.

In this study, we found that treatment with the lipid substrate-reducing compound Genz-123346, which inhibits glycosphingolipid biosynthesis, decreases mTORC1 activity, and restores TFEB expression in the *GBA1* mutant PD neurons. Previous studies reported similar positive effects of pharmacological restoration of lipid homeostasis on PD pathology. For example, inhibition of GlcCer synthase in primary neurons decreased AKT–mTOR signaling, increased autophagy flux, and reduced the mutant α-synuclein levels (Shen et al., 2014). Similarly, in a mouse model of *GBA*-PD, inhibition of glucosylceramide synthase reduced insoluble α-synuclein oligomerization and prevented the accumulation of ubiquitinated proteins (Sardi et al., 2017). Another study reported that inhibition of ceramide synthesis reduces α-synuclein aggregates and induces TFEB activation in a cellular model of PD (Mingione et al., 2021). It has also been shown that expression of wild-type *GBA1* successfully reduced lipid-rich α-synuclein aggregates and increased TFEB nuclear translocation in a PD mouse model (Glajch et al., 2021). Our results further confirm the pathogenic role of lipid accumulation on TFEB activity and identify mTOR signaling pathway as a key mediator of this process. Future studies are needed to explore the mechanisms of mTORC1 activation by sphingolipids in PD and the ability of the substrate reducing compounds to reverse proteinopathy manifestations. Emerging evidence indicates that TFEB plays a key role in maintaining cellular lipid homeostasis (Li et al., 2021) and therefore is involved in many diseases characterized by lipid imbalance (Li et al., 2021; Yan, 2022). Our study provides evidence that TFEB dysfunction might be mediated by pathogenic lipid accumulation in *GBA1*-associated PD and points to the potential therapeutic benefits of targeting mTORC1-TFEB axis on ameliorating the neurodegenerative process.

## Data availability statement

The original contributions presented in the study are included in the article/Supplementary material, further inquiries can be directed to the corresponding author.

## Author contributions

OA: study conceptualization. FM, AS, and OA: iPSC lines maintenance and differentiation. FM, AS, CS, NL, AB, and OA: data acquisition and analysis. ML, AB, AS, and OA: data interpretation and manuscript revision. FM, AS, and OA: manuscript preparation. All the authors read and approved the manuscript.

## Funding

This work was supported by grants from the National Institute of Neurological Disorders and Stroke (NINDS) 1R21NS123153-01, and The Maryland Stem Cell Research Fund (MSCRF) 2019-MSCRFD-5037 (to OA), and by the NINDS Human Cell and Data Repository (NHCDR) U24NS095914.

## Conflict of interest

The authors declare that the research was conducted in the absence of any commercial or financial relationships that could be construed as a potential conflict of interest.

## Correction note

A correction has been made to this article. Details can be found at: 10.3389/fnins.2025.1657693.

## Publisher’s note

All claims expressed in this article are solely those of the authors and do not necessarily represent those of their affiliated organizations, or those of the publisher, the editors and the reviewers. Any product that may be evaluated in this article, or claim that may be made by its manufacturer, is not guaranteed or endorsed by the publisher.
