# Correction: Deregulation of mTORC1-TFEB axis in human iPSC model of *GBA1*-associated Parkinson's disease

**DOI:** 10.3389/fnins.2025.1657693

**Published:** 2025-08-19

**Authors:** Fahad Mubariz, Afsoon Saadin, Nicholas Lingenfelter, Chinmoy Sarkar, Aditi Banerjee, Marta M. Lipinski, Ola Awad

**Affiliations:** ^1^Department of Microbiology and Immunology, University of Maryland School of Medicine, Baltimore, MD, United States; ^2^Department of Anesthesiology, University of Maryland School of Medicine, Baltimore, MD, United States; ^3^Department of Pediatrics, University of Maryland School of Medicine, Baltimore, MD, United States; ^4^Department of Anatomy and Neurobiology, University of Maryland School of Medicine, Baltimore, MD, United States

**Keywords:** *GBA1* mutations, Parkinson's disease, transcription factor EB, induced-pluripotent stem cells, autophagy-lysosomal pathway, mammalian target of rapamycin complex1

There was a mistake in Figure 1E as published. One of the fluorescence image panels in Figure 1E was accidently inserted twice. The marker expression image for VMAT2 in Ctrl^WT/WT^ was the same image shown for PD1-GBA^WT/N370S^ iPSC neuron population. The corrected Figure 1 appears below.

**Figure 1:**
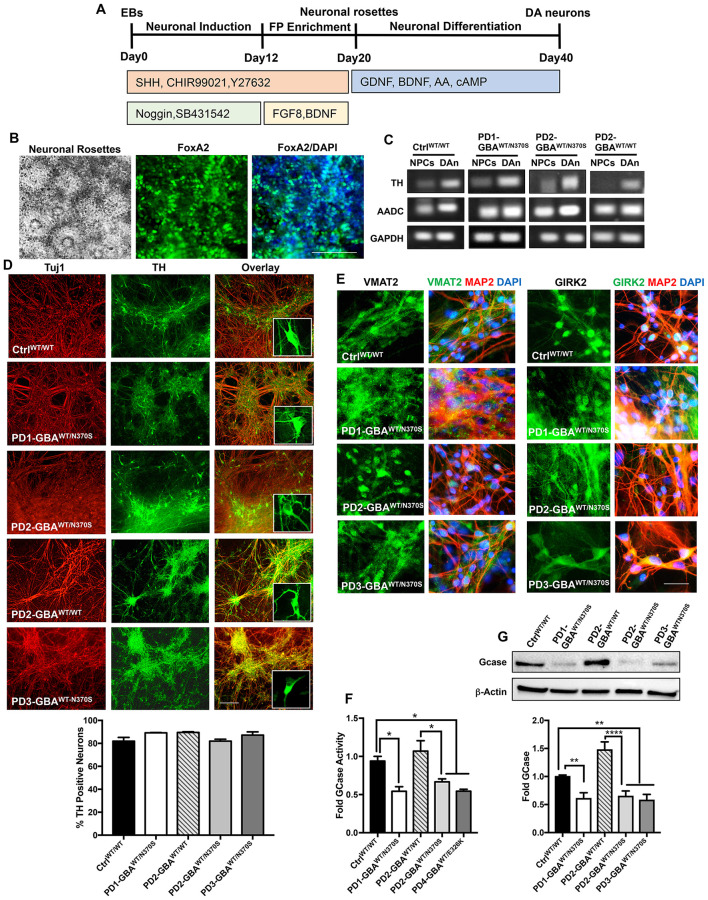
Generation of human iPSC model of *GBA1*-associated PD. **(A)** Schematic overview of the protocol used to differentiate iPSCs to dopaminergic (DA) neurons. Enrichment for midbrain floor-plate (FP) progenitors was initiated by culturing iPSCs-derived embryoid bodies (EBs) for 12 days in the presence of Noggin, SB431542 (SMAD signal inhibitors), CHIR99021 (WNT-β catenin signal activator), Sonic hedgehog (SHH) and Y27632 (ROCK inhibitor), followed by additional 8 days in the presence of FGF8 and BDNF. Neuronal rosettes appearing by day 20, were manually picked and differentiated to DA neurons by culturing in media supplemented with ascorbic acid (AA), cAMP, BDNF, and GDNF for 2–3 weeks. **(B)** Representative phase contrast image for iPSC-neuronal rosettes appearing around day 20 of the differentiation protocol showing the characteristic appearance. Also shown are immunofluorescence images of neuronal progenitors within the rosettes expressing the floor-plate marker FoxA2 and its overlay with nuclear DAPI. Scale bar = 100um. **(C)** Representative RT-PCR analysis showing expression of the dopamine synthesis enzymes; Tyrosine hydroxylase (TH), and Aromatic l-amino acid decarboxylase (AADC) in dopaminergic neurons (DAn) and neuronal progenitor cells (NPCs) differentiated from WT control (Ctrl ^WT/WT^), *GBA1* mutant, and gene-corrected PD iPSC lines. GAPDH is used as a loading control. **(D)** Representative immunofluorescence images of dopaminergic neuronal cultures (DNCs) differentiated from WT control and the indicated PD iPSC lines. Neurons were co-labeled with the pan-neuronal marker, Tuj1 (red) and the DA neuron marker, TH (green). Also shown in the last panels are the overlay of both markers and an enlargement of TH positive neurons in each line. Scale bar = 100 μm. Bar graph below shows the percentage of TH positive neurons in WT control and the indicated PD DNCs. Neurons were counted in at least 3 different fields per experiment in 2–3 independent experiments. Data represent average ± SEM. **(E)** Representative immunofluorescence images of WT control and the indicated *GBA1* mutant PD DNCs labeled with an antibody to the vesicular monoamine transporter 2 (VMAT2), or the G-protein regulated inward-rectifier potassium channel 2 (GIRK2). Also shown is the overlay of each marker (green) with MAP2 (red) and DAPI (blue). Scale bar = 50 μm. **(F)** GCase enzyme activity in WT control, *GBA1* mutant, and gene-corrected PD NPCs. Data represent average fold activity relative to control in triplicate wells in a representative experiment ± SEM. ^*^p = 0.02 between the indicated groups as assessed by One-way ANOVA. **(G)** Western blot analysis showing GCase protein levels in WT control, *GBA1* mutant, and gene-corrected PD DNCs. Also shown is β-Actin loading control. Bar graph shows fold GCase relative to the WT control. Data represent average ± SEM. *n* = 3–4 per group. ^**^p = 0.006 (WT vs. PD1), ^**^p = 0.008 (WT vs. PD2), ^**^p = 0.004 (WT vs. PD3), and ^****^p < 0.0001 between the indicated groups as assessed by One-way ANOVA.

The original version of this article has been updated.

