# Supplementary material for: Deregulation of mTORC1-TFEB axis in human iPSC model of GBA1-associated Parkinson’s disease
Source: Front Neurosci. 2023 Jun 2;17:1152503. doi: 10.3389/fnins.2023.1152503 (PMC10272450; doi:10.3389/fnins.2023.1152503)
Supplement: Supplementary file 1 [file Data_Sheet_1.docx]

Supplementary Material

**Deregulation of mTORC1-TFEB axis in human iPSC model of *GBA1*-associated Parkinson’s disease**

**Fahad Mubariz^1†^, Afsoon Saadin^1†^, Nicholas Lingenfelter^1^, Chinmoy Sarkar^2^, Aditi Banerjee^3^, Marta M. Lipinski^2,4^, and Ola Awad^1*^**

^†^ Authors contributed equally to work

*** Corresponding Author:** Ola Awad, PhD [oawad@som.umaryland.edu](mailto:oawad@som.umaryland.edu)


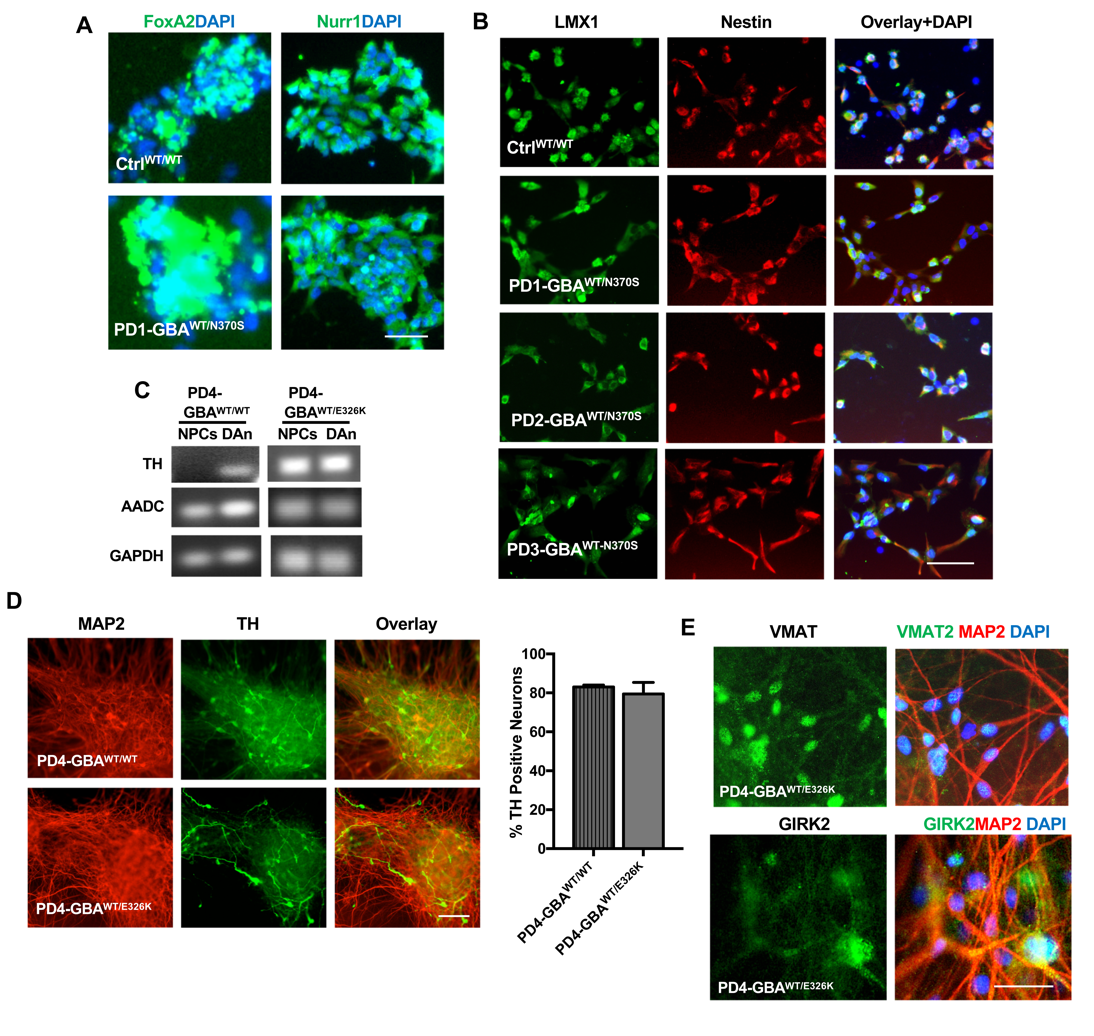


**Supplementary Figure 1. Characterization of** **the iPSC model of *GBA1*-associated PD*.* (A)** Representative immunofluorescence images for NPCs generated from WT control and the indicated *GBA1* mutant PD iPSC line showing the expression of the mid-floor plate marker FoxA2 or Nurr1 and its overlay with nuclear DAPI staining. Scale bar=50um.  **(B)** Representative immunofluorescence images for NPCs generated from WT control and the indicated *GBA1* mutant PD iPSCs lines. NPCs were co-labeled with antibodies against LMX1 (green) or Nestin (red), also shown is the overlay of both markers with nuclear DAPI staining. Scale bar=50um. **(C)** Representative RT-PCR analysis showing TH and AADC expression in NPCs and DA neurons differentiated from the indicated *GBA1* mutant and gene-corrected PD iPSC lines. GAPDH is used as a loading control. **(D)** Representative immunofluorescence images of DNCs differentiated from the indicated *GBA1* mutant and gene-corrected PD iPSC lines. Neurons were co-labeled with the pan-neuronal marker, MAP2 (red) and the DA neuron marker, TH (green). Also shown in the last panels is the overlay of both markers. Scale bar=100 μm. Bar graph shows the percentage of TH positive neurons in the indicated *GBA1* mutant and gene-corrected PD iPSC lines. Neurons were counted in at least 3 different fields in a representative experiment. Data represent average +SEM. **(E)** Representative immunofluorescence images of *GBA1* mutant PD DNCs labeled with an antibody to VMAT2 or GIRK2. Also shown is the overlay of each marker (green) with MAP2 (red) and DAPI (blue). Scale bar = 50 μm.

**
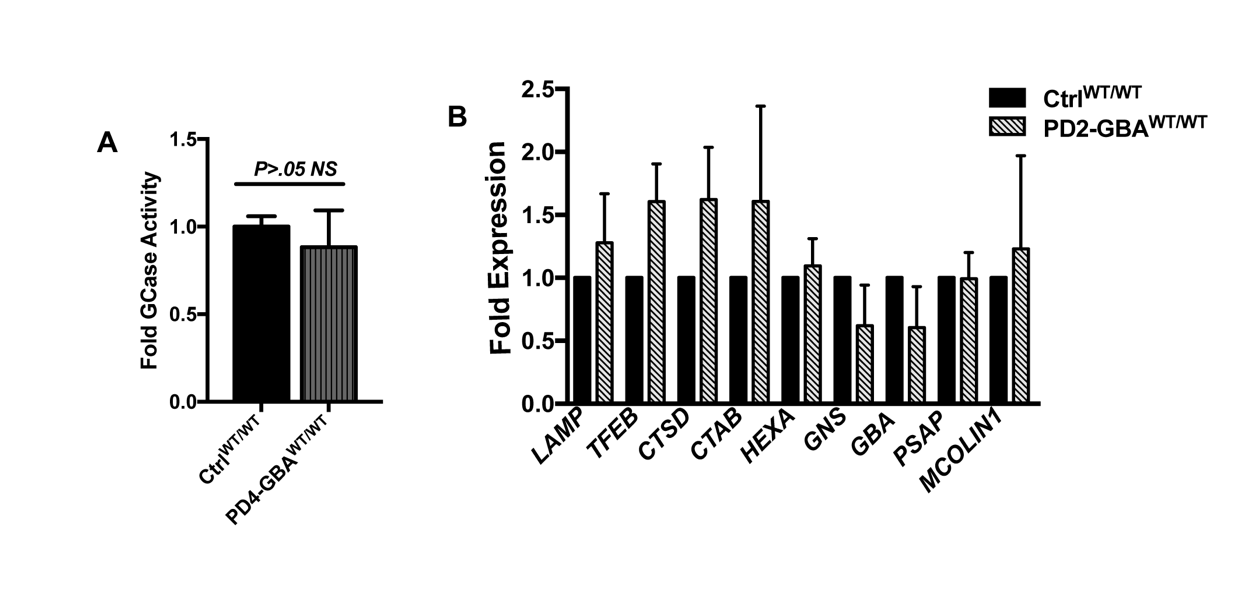
**

**Supplementary Figure 2. Functional characterization of the gene-corrected PD NPCs and DNCs*.* (A)** GCase enzyme activity in WT control, and gene-corrected PD NPCs. Data represent average fold activity relative to control in triplicate wells in a representative experiment ± SEM. *P*> 0.05, no significant difference (NS) between the indicated groups as assessed by Student’s *t*-test. **(B)** qRT-PCR analysis showing expression of TFEB target genes in WT control and gene-corrected PD DNCs. Data represent fold relative to control ±SEM in 3 independent experiments. *P*> 0.05 between WT control and gene-corrected cells for all genes examined as assessed by Student’s *t*-test.

**Supplementary Figure 3. Decreased TFEB levels in *GBA1* mutant PD neurons.** **(A)** Representative immunofluorescence images for WT control and *GBA1* mutant PD neurons labeled with anti-TFEB (green) and anti-Tuj1 (red) antibodies. Also shown in the last panel is the overlay of both markers and nuclear DAPI (blue). Scale bar=50 μm. **(B)** Western blot analysis for TFEB levels in *GBA1* mutant and gene-corrected PD DNCs. Also shown is β-Actin loading control. Bar graph represents fold TFEB relative to the gene-corrected cells ±SEM, *n* = 3 per group. **p*<0.05 as assessed by Student’s *t*-test.

**Supplementary Figure 4. Decreased nuclear TFEB expression in *GBA1* mutant PD neurons.** **(A)** Representative immunofluorescence images for WT control, *GBA1* mutant, and gene-corrected PD neurons labeled with anti-MAP2 (red) and anti-TFEB (green) antibodies. Also shown in the last panel is the overlay of both markers and nuclear DAPI (blue). Arrows point to nuclear TFEB fluorescence signal. Scale bar=25um. **(B)** Representative immunofluorescence images for gene-corrected and *GBA1* mutant PD neurons that was untreated or treated with 200 nM Torin1 for 18 hours. Cells were labeled with anti-MAP2 (red) and anti-TFEB (green) antibodies. Also shown in the last panel is the overlay of both markers and nuclear DAPI (blue). Arrows point to nuclear TFEB signal.


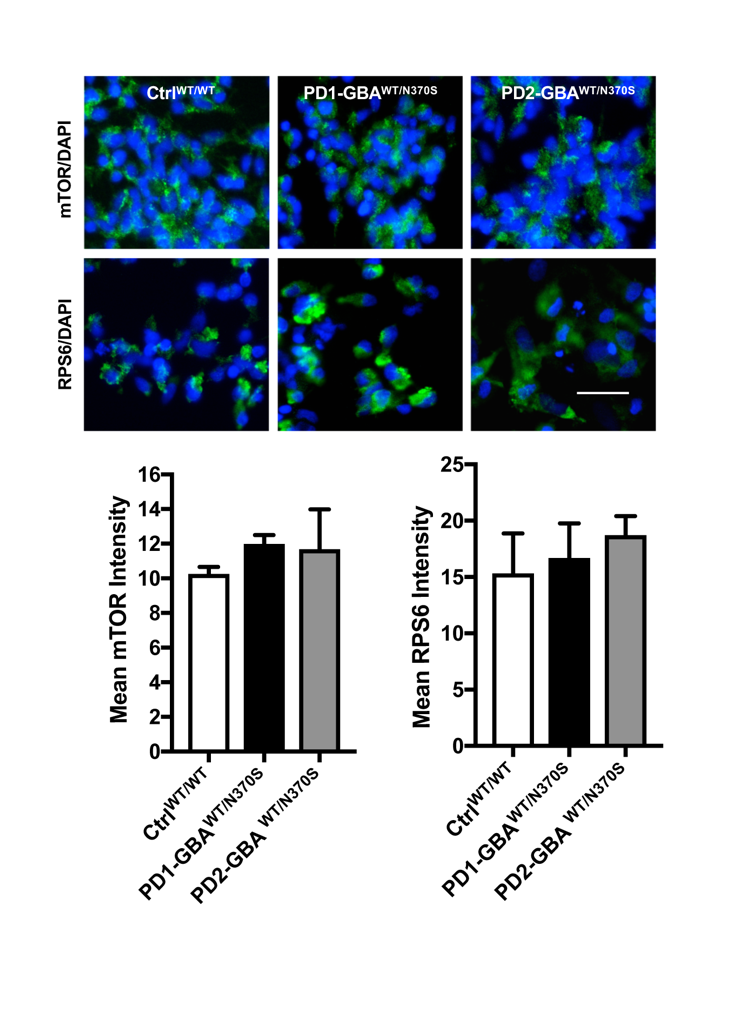


**Supplementary Figure 5. mTOR and RPS6 expression in iPSC-NPCs.** Representative immunofluorescence images of WT control and *GBA1* mutant NPCs labeled with antibodies to -mTOR or RPS6. Also shown is nuclear DAPI. Scale bar= 50 μm. Bar graphs below represent mean mTOR or RPS6 fluorescence signal intensity ±SEM. Data were collected from >30 cells per group, assayed in 3 different fields in a representative experiment. *P*> 0.05 between control and *GBA1* mutant NPCs as assessed by One-way ANOVA.

**Supplementary Figure 6. (A) Decreased DEPTOR levels in *GBA1* mutant PD neurons**. Western blot analysis for DEPTOR levels in *GBA1* mutant and gene-corrected PD DNCs. Also shown is β-Actin loading control. Bar graph represents fold relative to WT control. Data represent average ±SEM, *n* = 3 per group.***p*=0.004 (PD4 vs. PD4 corrected) and ***p*=0.003 (PD2 vs. PD2 corrected) as assessed by Student’s *t*-test. **(B) Effect of Torin on p-α-Synuclein (Ser 129) levels in gene-corrected DNCs**. Western blot analysis for p-α-Synuclein (Ser 129) levels in gene-corrected PD DNCs. Also shown is β-Actin loading control. Cells were either untreated or treated with 200 nM Torin1 for 18 hours. Bar graph represents p-α-Synuclein (Ser 129) fold relative to untreated cells ±SEM, *n* = 3 per group. *P*> 0.05 as assessed by Student’s *t*-test.

**Supplementary Table1: Sequence of RT-PCR primers.**

| AADC-F | 5′-GGGACCACAACATGCTGCTC-3′ |
| --- | --- |
| AADC-R | 5′-CCACTCCATTCAGAAGGTGCC-3′ |
| TH-F | 5-GGTTCCCAAGAAAAGTGTCAG-3′ |
| TH-R | 5′-GGTGTAGACCTCCTTCCAG-3′ |
| GAPDH-F | 5′-CAAGATCATCACGAATGC CTC-3′ |
| GAPDH-R | 5′-GCATGG ACTGTGGTCATGAGT C-3′ |

**Supplementary Table 2: Sequence of qRT-PCR primers.**

| TFEB-F | 5’-CCA GAA GCG AGA GCT CAC AGA T-3’ |
| --- | --- |
| TFEB-R | 5′- TGT GAT TGT CTT TCT TCT GCC G-3′ |
| LAMP1-F | 5′- ACG TTA CAG CGT CCA GCT CAT-3′ |
| LAMP1-R | 5′- TCT TTG GAG CTC GCA TTG G-3′ |
| HEXA-F | 5- CAA CCA ACA CAT TCT TCT CCA-3′ |
| HEXA-R | 5′- CGC TAT CGT GAC CTG CTT TT-3′ |
| CTSD-F | 5′- AAC TGC TGG ACA TCG CTT GCT-3′ |
| CTSD-R | 5′- CAT TCT TCA CGT AGG TGC TGG A-3′ |
| CTSB-F | 5’-AGT GGA GAA TGG CAC ACC CTA-3’ |
| CTSB-R | 5’-AAG AAG CCA TTG TCA CCC CA-3’ |
| CTSA-F | 5’-CAG GCT TTG GTC TTC TCT CCA-3’ |
| CTSA-R | 5’-TCA CGC ATT CCA GGT CTT TG-3’ |
| GBA-F | 5’-TGG GTA CCC GGA TGA TGT TA-3’ |
| GBA-R | 5’-AGA TGC TGC TGC TCT CAA CA-3’ |
| PSAP-F | 5’-GCC AAC AGT GAA ATC CCT TCC-3’ |
| PSAP-R | 5’-TCA GTG GCA TTG TCC TTC AGC-3’ |
| CLCN7-F | 5’- TGA TCT CCA CGT TCA CCC TGA-3’ |
| CLCN7-R | 5’-TCT CCG AGT CAA ACC TTC CGA-3’ |
| ATP6V1H-F | 5’-CAT TGT GAT GAG CGT GTT CTG G-3’ |
| ATP6V1H-R | 5’-AAC TCC CCG GTT AGG ACC CTT A-3’ |
| GNS-F | 5’-CCC ATT TTG AGA GGT GCC AGT-3’ |
| GNS-R | 5’-TGA CGT TAC GGC CTT CTC CTT-3’ |
| MCOLN1-F | 5’-TTG CTC TCT GCC AGC GGT ACT A-3’ |
| MCOLN1-R | 5’-GCA GTC AGT AAC CAC CAT CGG A-3’ |
| GAPDH-F | 5’-TGC ACC ACC AAC TGC TTA GC-3’ |
| GAPDH-R | 5’-GGC ATG GAC TGT GGT CAT GAG-3’ |
